# AMD1 upregulates hepatocellular carcinoma cells stemness by FTO mediated mRNA demethylation

**DOI:** 10.1002/ctm2.352

**Published:** 2021-03-24

**Authors:** Xinyu Bian, Dongmin Shi, Kailin Xing, Hongxin Zhou, Lili Lu, Dahai Yu, Weizhong Wu

**Affiliations:** ^1^ Liver Cancer Institute, Zhongshan Hospital, Fudan University, Key Laboratory of Carcinogenesis and Cancer Invasion Ministry of Education Shanghai China; ^2^ Department of Radiation Oncology Affiliated Hospital of Nanjing University of Chinese Medicine Nanjing Jiangsu China

**Keywords:** AMD1, FTO, hepatocellular carcinoma, IQGAP1, N6‐methyladenosine, polyamination, stemness

## Abstract

**Background:**

S‐adenosylmethionine decarboxylase proenzyme (AMD1) is a key enzyme involved in the synthesis of spermine (SPM) and spermidine (SPD), which are associated with multifarious cellular processes. It is also found to be an oncogene in multiple cancers and a potential target for tumor therapy. Nevertheless, the role AMD1 plays in hepatocellular carcinoma (HCC) is still unknown.

**Methods:**

HCC samples were applied to detect AMD1 expression and evaluate its associations with clinicopathological features and prognosis. Subcutaneous and orthotopic tumor mouse models were constructed to analyze the proliferation and metastasis of HCC cells after AMD1 knockdown or overexpression. Drug sensitive and tumor sphere assay were performed to investigate the effect of AMD1 on HCC cells stemness. Real‐time quantitative PCR (qRT‐PCR), western blot, immunohistochemical (IHC) and m6A‐RNA immunoprecipitation (Me‐RIP) sequencing/qPCR were applied to explore the potential mechanisms of AMD1 in HCC. Furthermore, immunofluorescence, co‐IP (Co‐IP) assays, and mass spectrometric (MS) analyses were performed to verify the proteins interacting with AMD1.

**Results:**

AMD1 was enriched in human HCC tissues and suggested a poor prognosis. High AMD1 level could promote SRY‐box transcription factor 2 (SOX2), Kruppel like factor 4 (KLF4), and NANOG expression of HCC cells through obesity–associated protein (FTO)‐mediated mRNA demethylation. Mechanistically, high AMD1 expression increased the levels of SPD in HCC cells, which could modify the scaffold protein, Ras GTPase‐activating‐like protein 1 (IQGAP1) and enhance the interaction between IQGAP1 and FTO. This interaction could enhance the phosphorylation and decrease the ubiquitination of FTO.

**Conclusions:**

AMD1 could stabilize the interaction of IQGAP1 with FTO, which then promotes FTO expression and increases HCC stemness. AMD1 shows prospects as a prognostic predictor and a therapeutic target for HCC.

## Graphical abstract

The interaction with IQGAP1 increases FTO phosphorylation and expression.

High level of FTO promotes pluripotency factors expression and elevates stem cell‐like property of HCC cells.

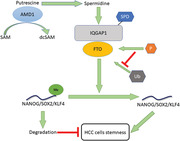

**Abbreviations**
AFP: alpha‐fetoprotein
ALDH: aldehyde dehydrogenase
ALKBH5: alkB homolog 5
AMD1: S‐adenosylmethionine decarboxylase proenzyme
CI: Confidence interval
Co‐IP: co‐immunoprecipitation
CSCs: cancer stem cells; EpCAM, epithelial cell adhesion molecule
ESCs: embryonic stem cells
FTO: obesity–associated protein
HCC: hepatocellular carcinoma
HCCDB: Integrative molecular database of hepatocellular carcinoma
HE: hematoxylin‐eosin
HR: hazard ratio
IHC: immunohistochemical
iPS: induced pluripotent stem cells
IQGAP1: Ras GTPase‐activating‐like protein 1
KLF4: Kruppel like factor 4
Me‐RIP: m6A‐RNA immunoprecipitation
METTL14: methyltransferase‐like 14
METTL3: Methyltransferase‐like 3
MS: mass spectrometric
OCT4: POU class 5 homeobox 1
PUT: Putrescine
RT‐qPCR: real‐time quantitative PCR
SOX2: SRY‐box transcription factor 2
SPD: spermidine
SPM: spermine
TMA: tissue microarray
WTAP: Wilms tumor 1 associated protein

## 1 BACKGROUND

Hepatocellular carcinoma (HCC) is the most frequent primary liver cancer and a leading cause of tumor‐associated mortality across the world. Although with the development in detection, diagnosis, and treatment, the majority of patients are at an advanced stage when diagnosticated, suffering postsurgical recurrence and having a low 5‐year survival rate.^1^, ^2^ Because of the lack of effective interventions and poor prognosis of recurrent HCC, a deep comprehension on the molecular mechanism of HCC recurrence is demanded imperiously.

Solid tumors and hematological malignancies often contain a small subpopulation of cells, which are named cancer stem cells (CSCs).^3^, ^4^ CSCs usually possess self‐renewal, multipotential differentiation capabilities and extensive proliferation, leading to drug resistance and tumor recurrence.^5^, ^6^ For HCC CSCs, many cell surface markers had been verified, including CD13, CD24, CD90 (THY1), CD 44, CD133, epithelial cell adhesion molecule (EpCAM) and aldehyde dehydrogenase (ALDH).^7^ Recent studies suggested that the expression of pluripotency factors, including NANOG, SRY‐box transcription factor 2 (SOX2), Kruppel like factor 4 (KLF4), and POU class 5 homeobox 1 (OCT4), could induce HCC cells reprogramming and self‐renewal as in the case of induced pluripotent stem cells.^8^, ^9^, ^10^ However, it is still obscure what biological roles CSCs play in HCC.

S‐adenosylmethionine decarboxylase proenzyme (AMD1) is a key enzyme involved in the synthesis of spermine (SPM) and spermidine (SPD).^11^ Polyamines have long been associated with cellular processes, including differentiation and cell proliferation.^12^ Polyamines can also be conjugated to proteins and may affect the protein–protein interaction and protein function.^13^ A recent study showed that AMD1 and its causal increase of polyamine synthesis could promote embryonic stem cells (ESCs) self‐renewal and maintain ESCs stemness by upregulating expression of multiple pluripotency factors.^14^, ^15^ AMD1 is also found to be an oncogene in multiple cancers and a potential target for tumor therapy.^16^, ^17^, ^18^ However, the function of AMD1 on HCC cells has not yet been reported. Thus, it is worth to catch a deep understanding of the molecular functions of AMD1 in HCC and develop more efficient targeted therapies for HCC.

Recent researches have suggested that pluripotency factors expressions were also regulated by their mRNA stability. N6‐methyladenosine (m6A) modification, the most predominant post‐translational modification of mRNA in mammalian cells, has been confirmed to be associated with mRNA stability and degradation.^19^, ^20^ This dynamic and reversible modification is catalyzed by two kinds of key catalytic proteins, which have seemingly opposite effects. Methyltransferase‐like 14 (METTL14) and methyltransferase‐like 3 (METTL3) compose the core methyltransferase complex with scaffold protein Wilms tumor 1 associated protein. Conversely, FTO and alkB homolog 5 (ALKBH5) reverse the methylation as demethylases.^21^, ^22^ Some pluripotency factors, including NONOG, SOX2, and KLLF4, were reported to be rich in m6A modification in ESCs.^19^ Besides, decreasing of m6A modification of NANOG mRNA led to improving its mRNA stability in both ESCs and breast CSCs (BCSCs).^23^ These results demonstrate that m6A methylation plays an important role in regulating stem cell stemness.

Here, we first investigated the role of AMD1 in HCC cells stemness and found that AMD1 regulated mRNA m6A modification by targeting FTO. Then we also demonstrated that SPD, the synthesized product modulated by AMD1, could modify IQGAP1 and enhance its interaction with FTO. This interaction leads to increase of FTO phosphorylation and decrease of FTO ubiquitination. Understanding mechanisms and functions of the AMD1‐IQGAP1‐FTO‐NANOG/SOX2/KLF4 axis may provide novel therapeutic strategies for HCC.

## 2 MATERIALS AND METHODS

### 2.1 Information of patients and follow‐up

Eighty‐five pairs of tumor and para‐tumor tissues from patients diagnosed with HCC undergoing hepatectomy in Zhongshan Hospital between January 2014 and April 2014 were collected. All tissues were embedded in paraffin and made into tissue microarray (TMA). None of them received any antitumor therapies before surgery. The follow‐up information was updated until January 2018. AMD1 and FTO expressions of TMA were quantified with mean density, which was measured and calculated as previously described.^24^ Additionally, 47 paired HCC and para‐tumor tissues were gathered and frozen for the following real‐time quantitative PCR (qRT‐PCR). All procedures met with approvals of the Research Ethics Committee of Zhongshan Hospital.

### 2.2 Cell culture

MHCC97H and HCCLM3 cell lines were previously established in the Liver Cancer Institute, Fudan University. PLC cell line was purchased from Chinese Academy of Sciences. All these cells were cultured in DMEM (Basalmedia, Shanghai, China) with 10% FBS (Gibco; NY, USA), and cultured with 5 % CO2 at 37°C.

### 2.3 Xenograft model in nude mice

Male BALB/c nude mice (4 weeks old) were obtained from Shanghai Laboratory Animal Co. Ltd. (Shanghai, China). Animal care followed the criteria in the “Guide for the Care and Use of Laboratory Animals”.

For subcutaneous xenotransplanted tumor models, cells were injected subcutaneously (5 × 10^6^ for MHCC97H or 1×10^6^ for PLC cells per mouse). Size of the tumors was measured every other day on Day 7 after injection. Relative tumor volumes were calculated as previously described.^25^ On Day 14, the mice were killed, and tumor tissues were collected for the following immunohistochemical (IHC) staining.

The orthotopic models were established as previously described.^26^ Five weeks later, transplanted mice were euthanized. Lung tissues were embedded in paraffin, sectioned serially, and stained with hematoxylin‐eosin (HE) staining for determining lung metastasis.

### 2.4 Cell transient transfections

FTO plasmid and the negative controls were obtained from GeneChem (Shanghai, China). The small interfering RNA against FTO (siFTO)/IQGAP1 (siIQGAP1) and the negative controls were obtained from Genepharma (Shanghai, China). Transient transfection was performed using Lipofectamine 3000 according to the product instruction transiently. The sequence of siRNA was presented in Table S1.

### 2.5 Construction of stable cell lines

AMD1 expression lentiviruses (pLenti‐EF1a‐EGFP‐P2A‐Puro‐CMV‐AMD1‐3Flag), AMD1 knockdown lentiviruses (pLKD‐CMV‐EGFP‐2A‐Puro‐U6‐AMD1 shRNA), and their corresponding negative controls were purchased from Oobio Co (Shanghai, China). The AMD1 expression lentiviruses were infected into PLC cells, while the knockdown lentiviruses were infected into MHCC97H and HCCLM3 cells, respectively. After infected, stably transfected cells were selected with puromycin (5 μg/ml, Sigma‐Aldrich, St. Louis, MO, USA).

### 2.6 RNA isolation and qRT‐PCR

Detailed procedures were conducted as described.^27^ GAPDH and 18sRNA were used as internal control for cells and tissues mRNAs assays, respectively. The primers of this study are shown in Table S1.

### 2.7 Measurement of mRNA stability

Cells were treated with vehicle or flavopiridol (Sigma‐Aldrich) at a concentration of 2μΜ for 3 h, followed by RNA isolation and qRT‐PCR.

### 2.8 Measurement of total m6A and m6A+ mRNA levels

The content of m6A in total RNA was analyzed with an m6A RNA methylation quantification kit (Epigentek, NY, USA) in accordance with the instruction. For measurement of m6A+ mRNA levels, RNA immunoprecipitation (RIP) was performed using the Magna MeRIP m6A Kit (Merck, Darmstadt, Germany) according to the manufacture protocol. The eluted RNA was used for cDNA synthesis and qRT‐PCR analysis.

### 2.9 Western blot assay

The procedures of western blot assay have been described previously.^27^ Antibodies that recognize AMD1 (11052‐1‐AP), Tubulin (11224‐1‐AP), NANOG (14295‐1‐AP), SOX2 (11064‐1‐AP), KLF4 (11880‐1‐AP), OCT4 (11263‐1‐AP), METTL3 (15073‐1‐AP), METTL14 (26158‐1‐AP), FTO (27226‐1‐AP), and ALKBH5 (16837‐1‐AP) were purchased from Proteintech (Wuhan, China). Antibodies that recognize IQGAP1 (ab86064), phosphoserine (ab9332), phosphothreonine (ab9338), and SPM (ab7318) were obtained from Abcam (Cambridge, MA, USA). Secondary antibodies were obtained from Beyotime Biotechnology (Shanghai, China).

### 2.10 Flow cytometric analysis

AMD1 expression/knockdown lentiviruses and their corresponding negative controls without fluorescence labeling were used for stable cell lines establishment. After transfected, cells were incubated with the following antibodies: APC‐CD90 (BioLegend, 328113) and PE‐CD44 (BioLegend, 103007). The subsequent steps were performed as previously described.^28^

### 2.11 IP and mass spectrometry assay

For IP, cell lysis was performed using Pierce IP Lysis Buffer (Thermo Fisher Scientific) in accordance with the instruction. Antibody against FTO (1:50, #31687, Cell Signaling Technology) or against IQGAP1 (1:100, ab86064, Abcam) was added to lysates with protein A/G beads and incubated overnight at 4℃. After centrifuged, the deposit was collected and washed with lysis buffer for three times. After resuspended in RIPA buffer, it was boiled for 5 min. The boiled compounds were centrifuged again, and the supernatant was used for following western blot and mass spectrometry (MS) assay. MS assay was to detect the potential interacting proteins as described previously.^29^ Open search strategy for higher confidence was applied to identify potential interacting proteins and various modifications as described.^30^

### 2.12 IHC staining

Immunohistochemistry was performed on TMA and tumor tissues from subcutaneous transplanted models. The sections were hybridized with primary antibody against AMD1 (1:200), FTO (1:200), NANOG (1:100), SOX2 (1:200), and KLF4 (1:200). Then, the following procedures were performed using GTVision III detection system kit (Gene Tech, Shanghai, China) according to the manufacture protocol.

### 2.13 Immunofluorescence

PLC^AMOE^ cells were cultured on confocal dishes for 24 h. For double immunofluorescence staining, cells were incubated with FLAG antibody (#8146, Cell Signaling Technology) and FTO antibody (abs110820, Absin) at 4°C overnight after fixated with 4% paraformaldehyde and permeabilized with 0.2% Triton X‐100. Then, the cells were incubated with goat anti‐rabbit‐conjugated antibodies (A0408, Beyotime) and anti‐mouse‐conjugated antibodies (A0473, Beyotime) at room temperature for 30 min in the dark. At last, nucleus as counterstained with DAPI. Extra antibodies for triple immunofluorescence included FLAG antibody (ab1257, abcam) and IQGAP1 antibody (H00008826‐M01, Abnova).

### 2.14 Drug sensitive, tumor sphere, and clone formation assay

For drug sensitive test, cells (2 × 10^3^ per well) were seeded into a 96‐well plate with 200 μl DMEM with different concentrations of sorafenib (0 μM to 11 μM consecutively) and incubated for 72 h. Then the CCK8 assay was performed, and IC50 value was calculated with iC50‐calculator.

Tumor sphere assay was performed as previously reported.^27^ After 7 days, spheres with diameter > 50μm were counted, and the rate of spheres formation was calculated by the following formula: rate of spheres formation = the amounts of spheres/500 × 100%. The cells spheres were collected for flow cytometric analysis.

For clone formation assay, 1000 cells were seeded in a 60 mm culture dish and cultured for 2 weeks. Then, cells were stained with crystal violet. The numbers of visible colonies were calculated microscopically.

### 2.15 Bioinformatics prediction and database analysis

The overall survival (OS) and relapse‐free survival (RFS) of HCC patients were also analyzed by HCCDB (Integrative molecular database of hepatocellular carcinoma, http://lifeome.net/database/hccdb/home.html) and Kaplan‐Meier plotter (http://kmplot.com/analysis/). AMD1 mRNA levels in different tumors were analyzed by UALCAN (http://ualcan.path.uab.edu/index.html). The m^6^A sites were predicted via SRAMP (http://www.cuilab.cn/sramp). IC50 value was calculated with ic50‐calculator (https://www.aatbio.com/tools/ic50‐calculator). Processed data of GSE14520 were downloaded from GEO database.

### 2.16 Statistical analysis

Data were analyzed using SPSS 24.0. Quantitative variables were revealed as means ± SD. Differences between two groups were compared by Student's *t*‐test. The correlations between AMD1 and the clinical and pathological features were analyzed using the chi‐square test. Pearson's correlation analysis was performed between AMD1 and FTO expression. OS and disease‐free survival (DFS) were analyzed using the Kaplan–Meier method. The Cox hazard regression model was performed for univariate and multivariate analyses. *p*‐values < 0.05 were considered significant.

## 3 RESULTS

### 3.1 Clinical significances of AMD1 in HCC patients

**FIGURE 1:**
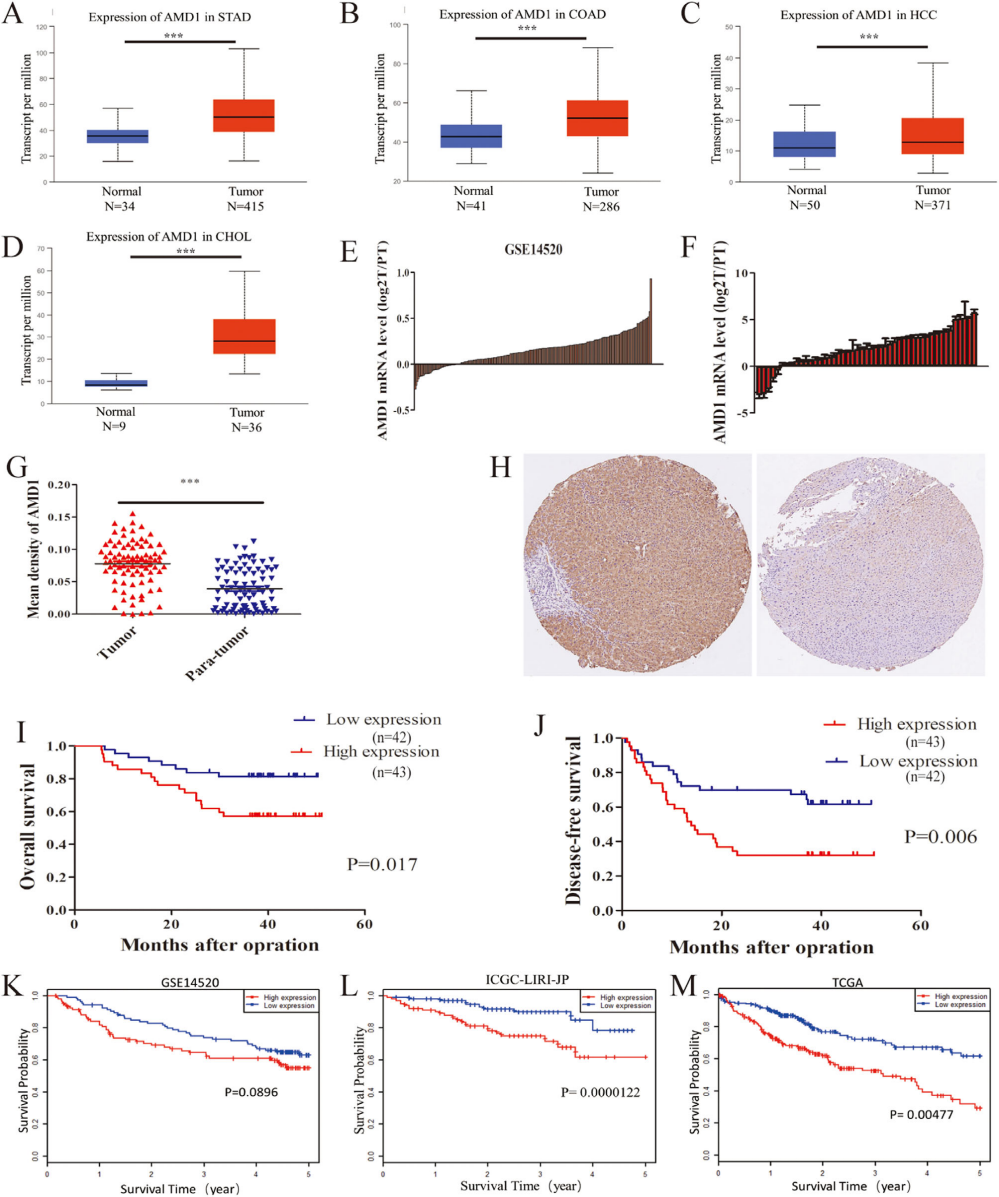
AMD1 expression is upregulated in HCC and predicts poor prognosis. (A‐D) AMD1 mRNA levels in different cancers were analyzed by UALCAN, ***p < 0.001. (E) The mRNA expression of AMD1 of 233 paired tumors and para‐tumor tissues in GSE14520 cohorts. (F) The mRNA level of AMD1 in HCC tissues compared with tumor tissues (T) and para‐tumor tissues (PT) in our study. (G) AMD1 expression in 85 paired HCC tissues and para‐tumor tissues from the immunohistochemistry results, ***p < 0.001. (H) Representative images of AMD1 expression in HCC tissues and adjacent para‐tumor liver tissues. (I and J) Kaplan–Meier analysis indicates high expression of AMD1 is correlated with poor overall survival and disease‐free survival (*n* = 85). (K‐M) Overall survival analysis of HCC patients according to AMD1 expression in GSE14520, ICGC‐LIRI‐JP, and TCGA cohorts performed by HCCDB Abbreviations: CHOL, cholangio carcinoma; COAD, colon adenocarcinoma; HCC, hepatocellular carcinoma; PT, para‐tumor tissues; STAD, stomach adenocarcinoma; T, tumor tissues.

By retrieving the database, we noticed that AMD1 mRNA levels were upregulated in multiple alimentary system cancers, including HCC (Figures 1A‐1E). In our study, similar result was observed in 47 paired HCC tumor and para‐tumor tissues by qRT‐PCR technique (Figure 1F). Further, TMA was stained by immunohistochemistry to verify the clinical significances of AMD1 in HCC. As shown in Figures 1G and 1H, AMD1 expression of tumor tissues was also significantly higher than their paired para‐tumor tissues in protein level (*p* < 0.0001). Further, AMD1 expression was significantly associated with tumor size (*p* = 0.037) and preoperative serum alpha‐fetoprotein (AFP) level (*p* = 0.038, Table 1). The Kaplan–Meier survival results revealed patients with high AMD1 expression had a shorter OS (*p* = 0.017) and DFS (*p* = 0.006, Figures 1I and 1J). The survival analysis of GSE14520, ICGC‐LIRI‐JP, and TCGA cohorts showed the same trend (Figures 1K‐1M). As shown in Tables 2 and 3, univariate and multivariate survival analysis indicated that AMD1 expression was an independent poor prognostic factor for OS (*p* = 0.002, hazard ratio (HR） = 4.043, 95% Confidence interval (CI) = 2.093‐23.062) and DFS (*p* = 0.004, HR = 2.550, 95% CI = 1.359‐4.787). Taken together, these results suggest the AMD1 expression may have a significant effect on the prognosis of HCC patients.

**TABLE 1:** Correlation between the clinicopathologic characteristics and AMD1 expression in HCCs (*n* = 85)

Variable	Case	AMD1 expression		
		Low (43)	High (42)	*p*‐value
Gender				0.386
Male	68	36	32	
Female	17	7	10	
Age (years)				0.591
≤60	57	30	27	
>60	28	13	15	
HBSAg				0.391
Negative	13	8	5	
Positive	72	35	37	
AFP (ng/ml)				**0.038**
≤20	50	30	20	
>20	35	13	22	
Tumor size (cm)				**0.037**
Size ≤ 3	14	11	3	
3 > size ≤ 5	18	6	12	
size > 5	53	26	27	
Tumor number				0.867
Solitary	60	30	30	
Multiple	25	13	12	
Tumor differentiation				0.160
I and II	45	26	19	
III and IV	40	17	23	
Microvascular invasion				0.929
Absent	51	26	25	
Present	34	17	17	
Tumor encapsulation				0.867
Absent	25	13	12	
Present	60	30	30	
BCLC staging system				0.088
0 and A	32	20	12	
B and C	53	23	30	

**TABLE 2:** Univariate and multivariate analyses of risk factors associated with overall survival of 85 HCC cases

	Overall survival			
	Univariate analysis		Multivariate analysis	
Variable	HR (95% CI)	*p‐*value	HR (95% CI)	*p*‐value
Gender		0.938		NA
Male vs. female				
Age (years)		0.063		NA
≤50 vs.>50				
HBSAg		0.590		NA
Negative vs. positive				
AFP (ng/ml)		0.423		NA
≤20 vs.>20				
Tumor size (cm)	2.875 (1.280‐6.459)	**0.011**	2.851 (1.081‐7.520)	**0.034**
≤3 vs.3‐5 vs. > 5				
Tumor number	2.238 (1.027‐4.848)	**0.043**	3.469 (1.410‐8.538)	**0.007**
single vs. multiple				
Tumor differentiation	2.627 (1.169‐5.501)	**0.015**		0.381
I and II vs. III and IV				
Microvascular invasion	2.444 (1.121‐5.327)	**0.025**		0.999
Absent vs. present				
Tumor encapsulation	4.595 (2.068‐10.064)	**0.000**	8.699 (2.903‐26.062)	**0.000**
Absent vs. present				
AMD1 expression	2.646 (1.150‐6.088)	**0.022**	4.043 (2.093‐23.062)	**0.002**
Low vs. high				

**TABLE 3:** Univariate and multivariate analyses of risk factors associated with disease‐free survival of 85 HCC cases

	Disease‐free survival			
	Univariate analysis		Multivariate analysis	
Variable	HR (95% CI)	*p*‐value	HR (95% CI)	*p*‐value
Gender		0.382		NA
Male vs. female				
Age (years)		0.158		NA
≤60 vs.>60				
HBSAg		0.640		NA
Negative vs. positive				
AFP (ng/ml)		0.412		NA
≤20 vs.>20				
Tumor size (cm)	1.915 (1.181‐3.105)	**0.008**		0.142
≤3 vs. 3‐5 vs. < 5				
Tumor number	2.076 (1.129‐3.819)	**0.019**	2.146 (1.097‐4.201)	**0.026**
single vs. multiple				
Tumor differentiation		0.096		
I and II vs. III and IV				
Microvascular invasion	2.081 (1.149‐3.771)	**0.016**		0.543
Absent vs. present				
Tumor encapsulation	2.402 (1.309‐4.407)	**0.005**	2.665 (1.410‐5.039)	**0.003**
Absent vs. present				
AMD1 expression	2.317 (1.249‐4.297)	**0.008**	2.550 (1.359‐4.787)	**0.004**
Low vs. high				

### 3.2 AMD1 enhanced HCC proliferation and metastasis in vivo

**FIGURE 2:**
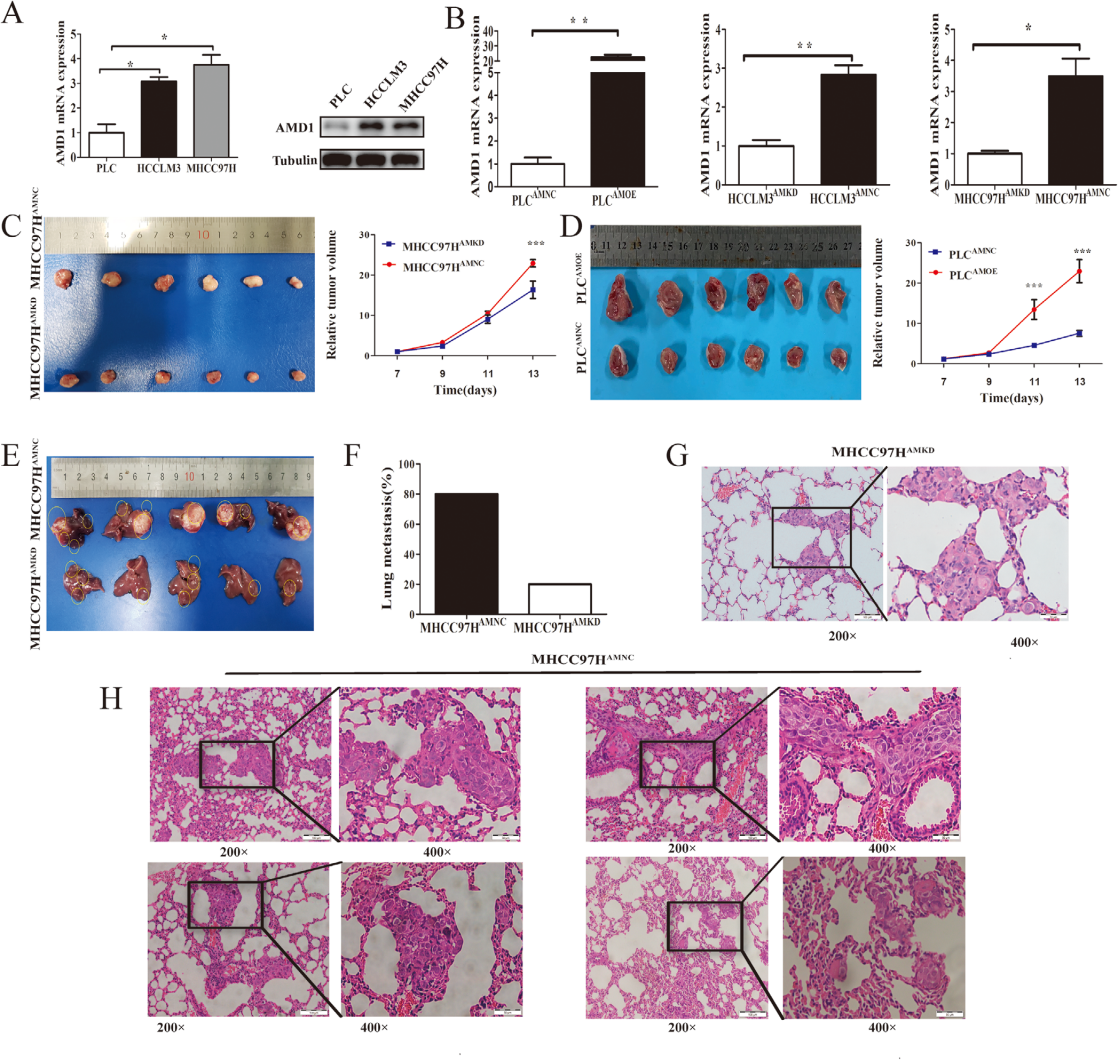
Downregulation of AMD1 inhibits HCC growth and metastasis in vivo. (A) Real‐time PCR and western blot and analysis of the AMD1 mRNA and protein expression in different HCC cells. *p < 0.05. (B) The mRNA levels of transcription factors in HCC cells infected with AMD1 overexpression or knockdown and negative control lentivirus. *p < 0.05, **p < 0.005. (C and D) Macrograph and absolute volume of subcutaneous xenograft tumors. ***p < 0.001. (E) Macrograph volume of orthotopic xenograft tumors. (F) The percentage of mice with metastasis in the lungs. (G and H) Representative pictures for lung metastasis of each mouse

After stable cells lines were established with of HCC cells of differing metastatic potential (Figures 2A and 2B), subcutaneous and orthotopic xenograft model in nude mouse were applied to evaluating the function of AMD1 in vivo. As expected, AMD1 knockdown in MHCC97H cells decreased the tumor volume and growth rate compared to controls. The same trend was observed in PLC cells groups (Figures 2C‐2E). Meanwhile, the pulmonary metastasis rate of control group was 80% (4/5), whereas 20% (1/5) lung metastasis was found in MHCC97H^AMKD^ group (Figures 2F‐2H). These consequences indicated that AMD1 could promote both HCC cells growth and metastasis in vivo.

### 3.3 AMD1 increased stem cell‐like property and of HCC cells

**FIGURE 3:**
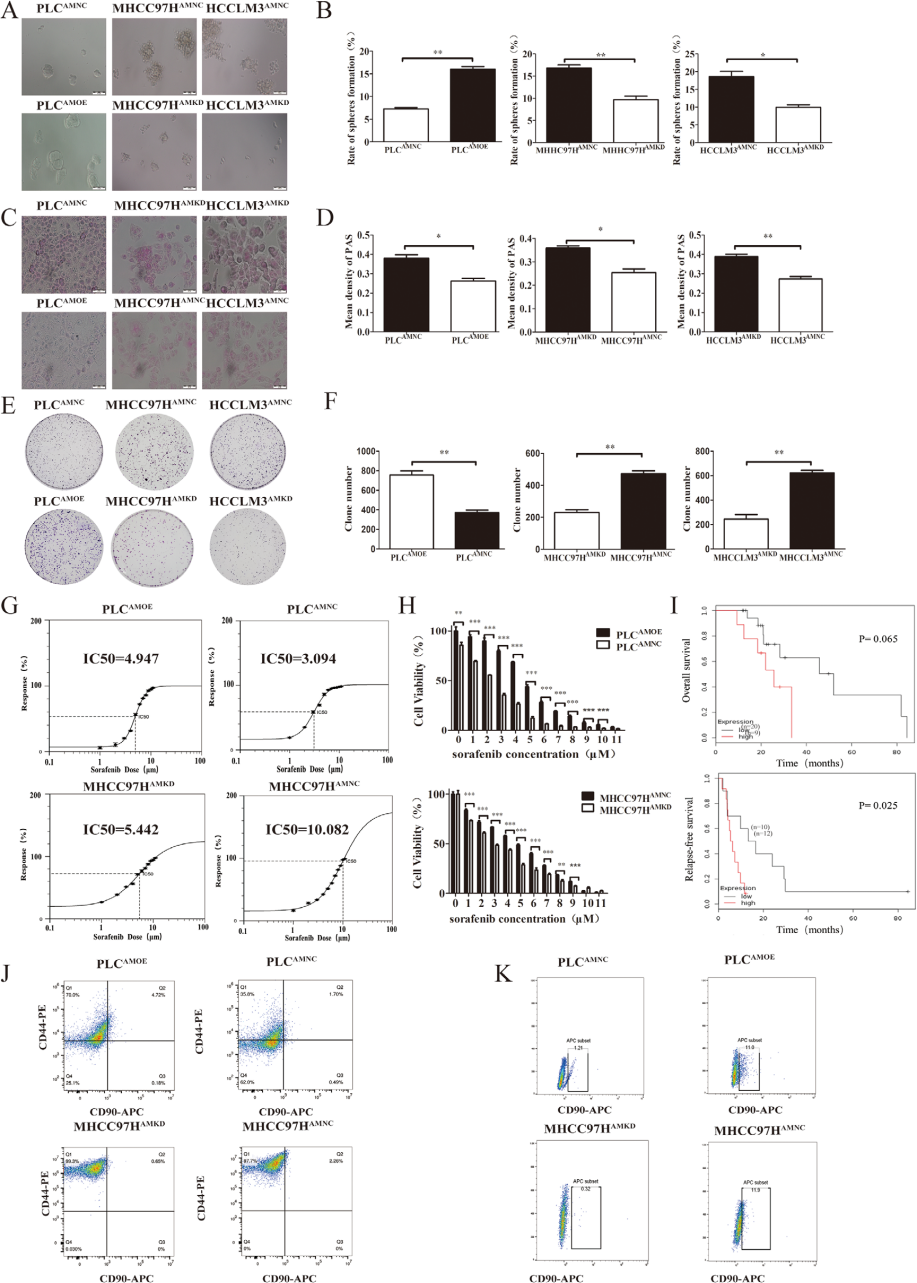
AMD1 promotes the stemness of HCC cells. (A and B) The effects of AMD1 overexpression or knockdown on sphere‐forming ability detected by sphere‐forming assays. *p < 0.05, **p < 0.005. (C and D) The effects of AMD1 overexpression or knockdown on glycogen content in HCC cells examined by PAS stain. *p < 0.05, **p < 0.005. (E and F) Representative images and statistical results of PLC, MHCC97H and MHCCLM3 cells clone formation assay after transfected with corresponding vectors. **p < 0.01 (G) The IC50 and growth‐inhibitory curves of AMD1 overexpression or knockdown in HCC cells were assayed by ic50‐calculator. (H) CCK8 assay was performed to detect cell proliferation after treatment with sorafenib for 72 h. **p < 0.01, ***p < 0.001. (I) Subgroup analysis of TCGA cohort from Kaplan‐Meier plotter revealed that patients with low AMD1 expression treated with sorafenib had better clinical outcomes. (J) Flow cytometry using antibodies specifically targeting CD44 and CD90 detected an enrichment of the CD90+CD44+ cells. (K) Flow cytometry detected an enrichment of the CD90+ cells after sphere‐forming assay

To determine whether AMD1 affected HCC stemness as well, we performed tumor sphere formation assay in AMD1 overexpressed or downregulated HCC cells. Indeed, the formation rate of tumor spheres was significantly increased in PLC^AMOE^ cells than those in control groups. Similar results were observed in MHCC97H and HCCLM3 cells (Figures 3A and 3B). The glycogen content of liver cells can reflect the differentiation degree, which often declines gradually during carcinogenesis.^31^ Periodic Acid‐Schiff stain revealed that glycogen was decreased in PLC^AMOE^ cells compared with PLC^AMNC^ cells, while it was increased in MHCC97H^AMKD^ and HCCLM3^AMKD^ compared to controls (Figures 3C and 3D). The results of plate clone formation assay indicated that upregulation of AMD1 in PLC cells increased cell colony‐formation ability, whereas knockdown of AMD1 in MHCC97H and MHCCLM3 cells reduced this potential compared with their corresponding control group cells (Figures 3E and 3F). Because drug resistance is a key feature of CSCs, we therefore evaluated whether high AMD1 expression could increase HCC cells drug resistance. AMD1 overexpressed or downregulated HCC cells, and their control groups were treated with sorafenib for 72 h before CCK8 assay was performed. Then the inhibitive concentration (IC50) of sorafenib of each cell lines was calculated. High level of AMD1 could protect cells from sorafenib toxicity and increase the IC50 values of HCC cells (Figures 3G‐3H). Besides, the subgroup analysis of TCGA cohort revealed that when treated with sorafenib, patients with low AMD1 levels had a longer OS and RFS (Figure 3I). CD90 and CD44 were reported as surface marker of HCC CSCs. CD44^+^CD90^+^ cells were more aggressive and had stronger tumor‐forming capacity.^28^ Predictably, overexpression of AMD1 in PLC cells elevated the proportion of CD44^+^CD90^+^ cells (4.56 ± 0.15% vs. 1.95 ± 0.21%, *p* < 0.001), whereas AMD1 down‐regulation in MHCC97H cells reduced the double positive cells rate (Figure 3J, 2.18 ± 0.12% vs. 0.8 ± 0.24%, *p* < 0.05). Cells selected from the tumor spheres were also detected with flow cytometric analysis. High AMD1 expression in PLC cells elevated the proportion of CD90+ cells (12.5 ± 1.35% vs. 2.07 ± 0.81%, *p* < 0.005), whereas knockdown of AMD1 reduced the positive cells rate in MHCC97H cells (10.0 ± 1.78% vs. 1.03 ± 0.63%, *p* < 0.05, Figure 3K). These results suggested that AMD1 could increase HCC cells stem cell‐like property.

### 3.4 AMD1 promoted pluripotency factors expression of HCC cells

**FIGURE 4:**
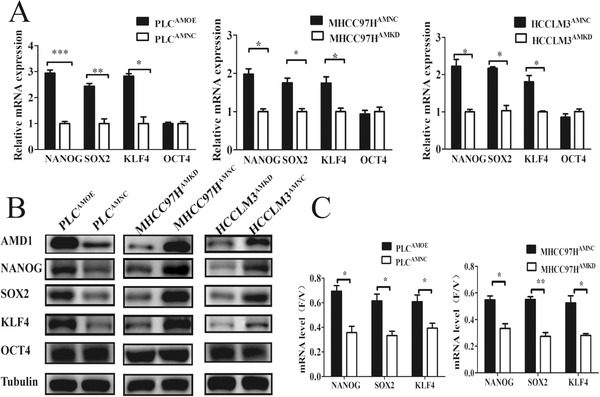
AMD1 regulates pluripotency factors expression of HCC cells. (A and B) The mRNA and protein levels of transcription factors in HCC cells infected with AMD1 overexpression or knockdown and negative control lentivirus. *p < 0.05, **p < 0.005, ***p < 0.001. (C) AMD1 increased NANOG, SOX2, and KLF4 mRNA stability in HCC cells. *p < 0.05, **p < 0.005

To identify the underlying mechanism, the expression of four core transcriptional factors, NANOG, SOX2, KLF4, and OCT4, which are major regulators of CSCs self‐renewal and pluripotency maintaining were examined. Increasing AMD1 level in PLC cells could significantly upregulate NANOG, SOX2, and KLF4 expression except for OCT4, whereas decreasing AMD1 levels in MHCC97H and HCCLM3 cells could downregulate NANOG, SOX2, and KLF4 expression (Figures 4A and 4B).

Both increase of transcription and decrease of degradation could raise the levels of mRNAs, then improving protein expression.^32^ Thus, we measured the mRNA stability of these transcriptional factors as described previously.^23^ After treatment for 2 h, the ratio of mRNA of cells treated with flavopiridol relative to control groups treated with vehicle only (F/V ratio) was calculated. Increased expression of AMD1 could promote F/V rations of mRNA of NANOG, SOX2, and KLF4 (Figure 4C), which meant a decreasing degradation of targeted mRNA. These data suggested that AMD1 played a promoting role on HCC CSCs‐like properties via upregulating expression of NANOG, SOX2, and KLF4.

### 3.5 AMD1 regulates m6A methyladenosine modification of HCC cells via FTO

**FIGURE 5:**
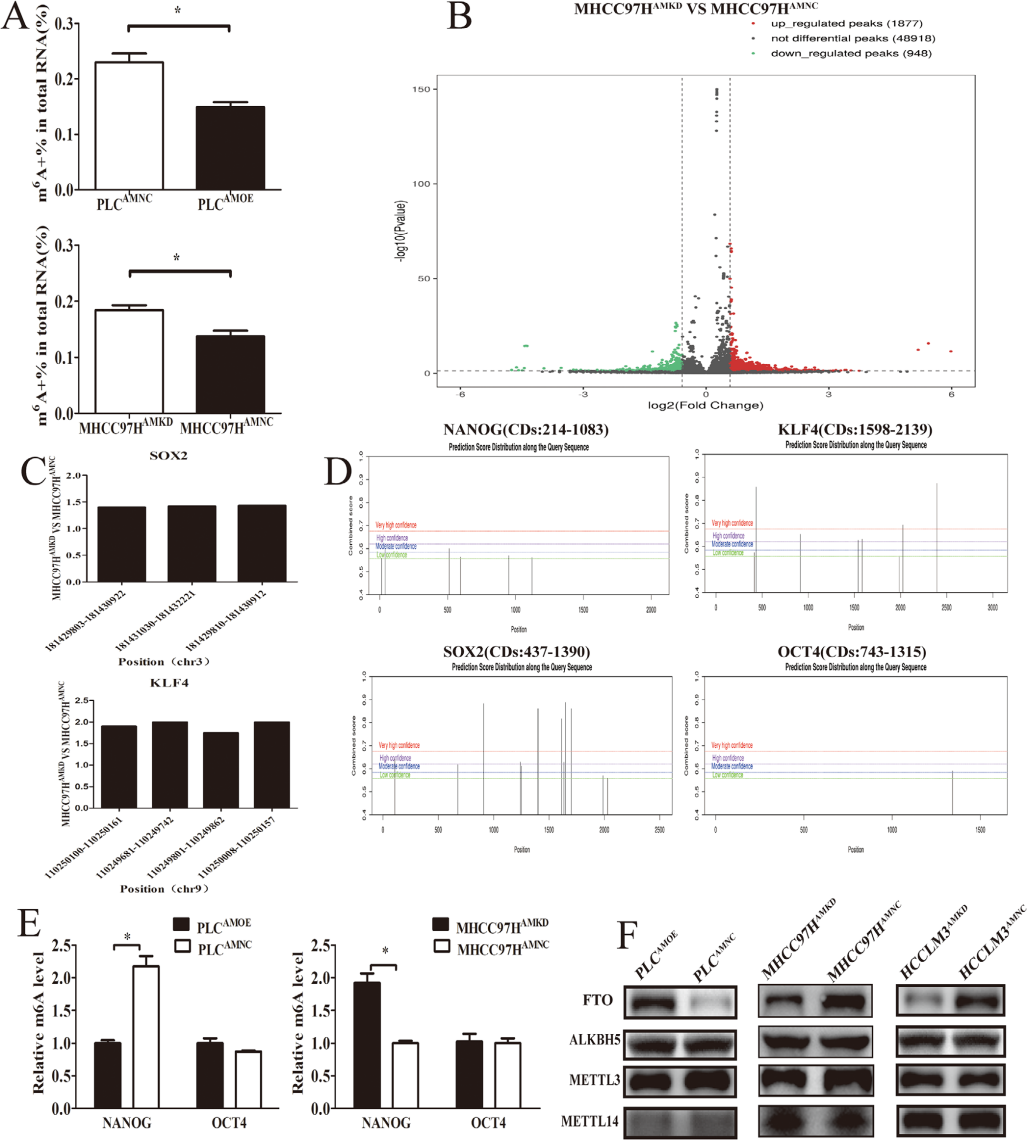
AMD1 regulates m6A methyladenosine modification of HCC cells. (A) Overexpression of AMD1 decreased m6A content in PLC cells, while knockdown of AMD1 increased m6A content in MHCC97H cells. *p < 0.05. (B) The significantly increased (red) or decreased (green) m6A peaks (*p* < 0.05) in MHCC97HAMKD cells compared with MHCC97HAMNC cells. (C) m6A abundance in modification sites of SOX2 and KLF4 mRNA identified with m6A‐Seq in MHCC97HAMKD cells compared with MHCC97HAMNC cells. (D) m6A sites were predicted in NANOG, SOX2, KLF4, and OCT4 with SRAMP. (E) Gene‐specific m6A qPCR analysis of alterations in the m6A level in NANOG CDs and OCT4 3′UTR regions in HCC cells with different AMD1 levels. *p < 0.05. (F) The m6A methyltransferase/demethylase expression in PLC and MHCC97H cells transfected with AMD1 overexpression or knockdown and the negative control vectors

**FIGURE 6:**
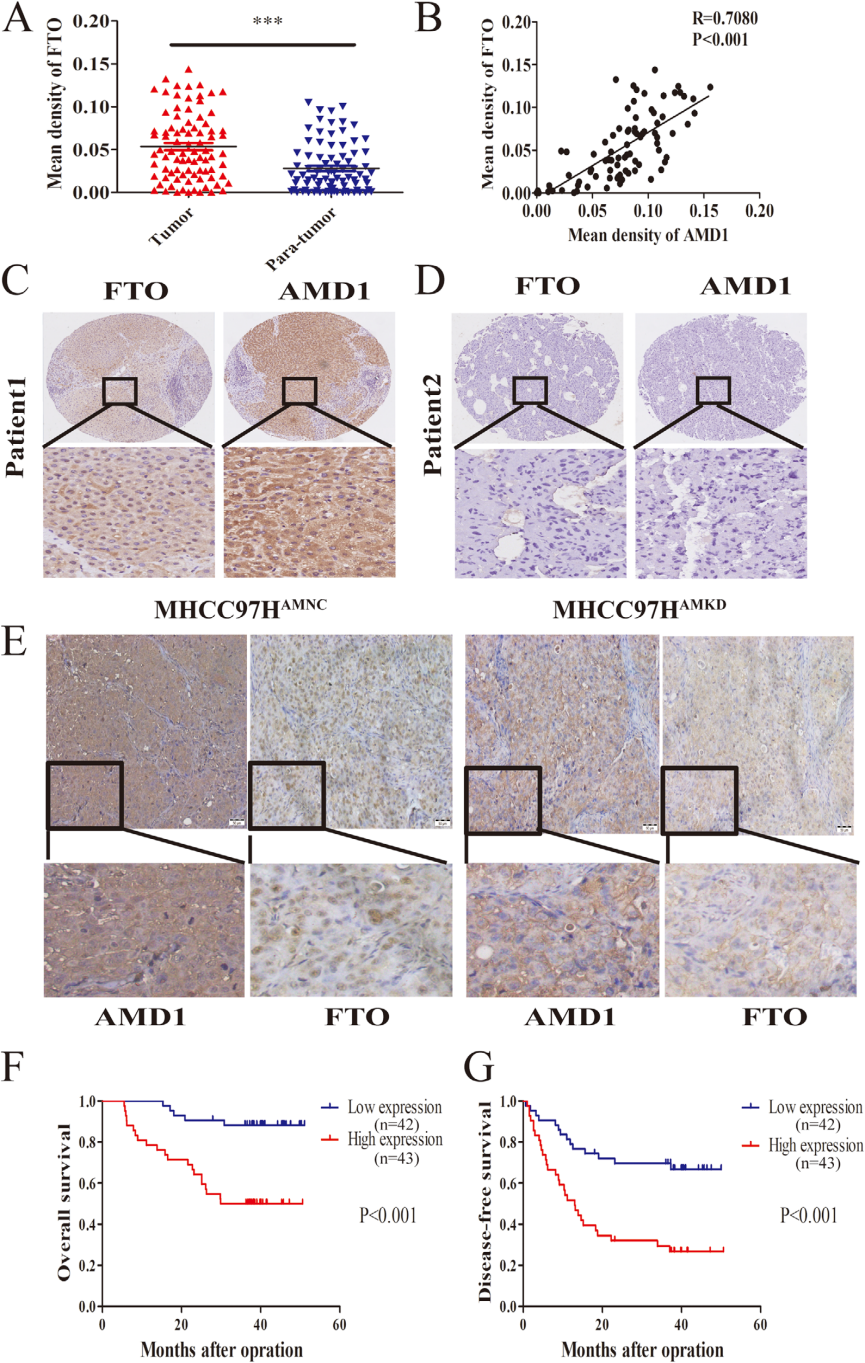
AMD1 promotes HCC stemness through FTO. (A) FTO expression in 85 paired HCC tissuesand para‐tumor tissuesfrom the immunohistochemistry results. ***p < 0.001. (B) Positive correlation between AMD1 and FTO in HCC tissues from TMA (*n* = 85). (C and D) Representative images of IHC staining of FTO in tumor tissues of HCC patients with high or low level of AMD1 expression. (E) Representative immunohistochemical staining of AMD1 and FTO of subcutaneous xenograft tumors (magnification 200×, Scale bar: 50 μm). (F and G) Kaplan–Meier analysis indicates high expression of FTO is correlated with poor overall survival and disease‐free survival (*n* = 85)

**FIGURE 7:**
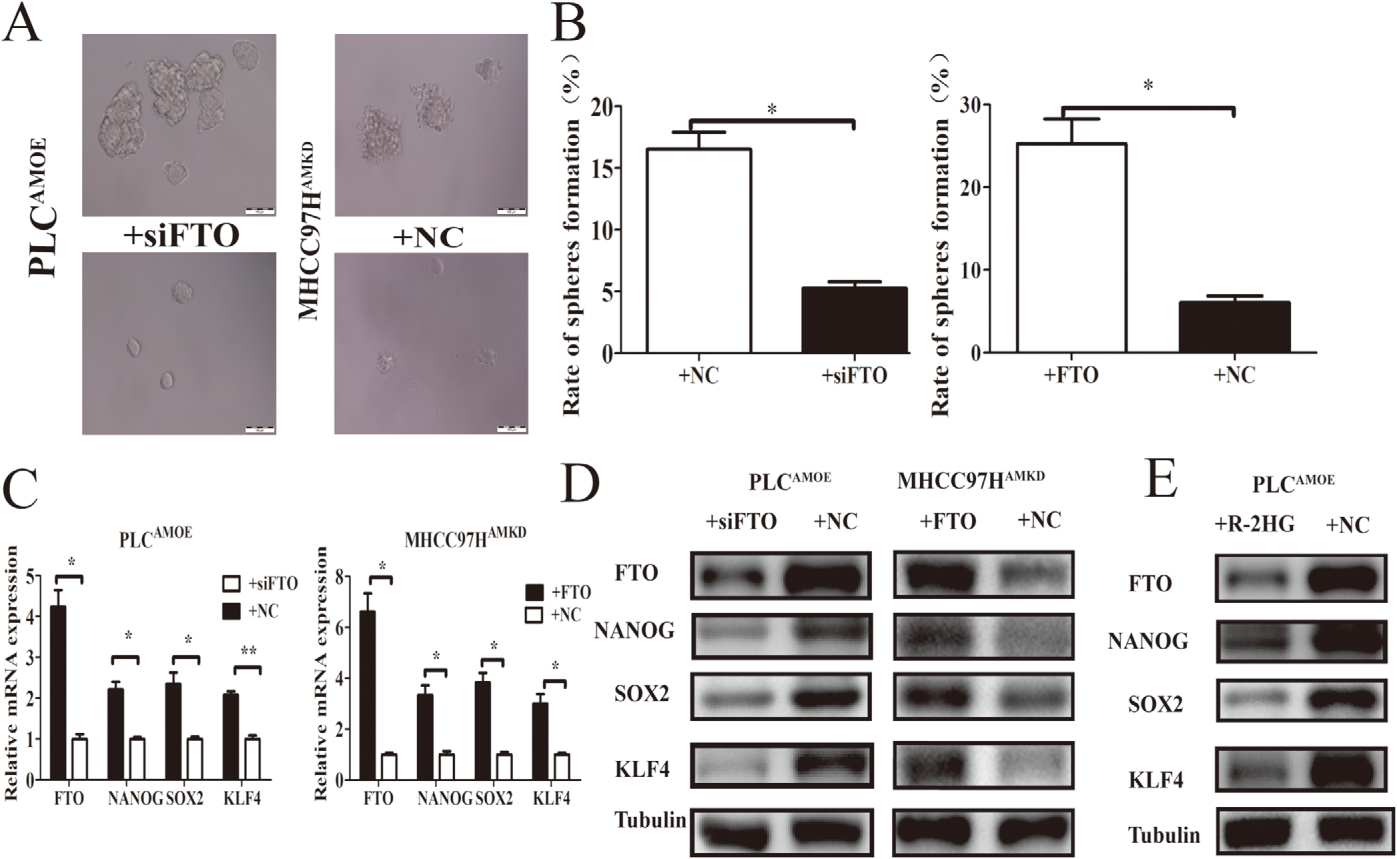
FTO acted as a functional downstream target of AMD1. (A and B) FTO knockdown in PLC^AMOE^ cells decreased the sizes and formation rate of tumor spheres, while FTO restoration in MHCC97H^AMKD^ cells increased the sizes and formation rate of tumor spheres. *p < 0.05. (C and D) FTO knockdown in PLC^AMOE^ cells decrease transcription factors expression while FTO restoration in MHCC97H^AMKD^ cells promotes transcription factors expression. *p < 0.05, **p < 0.005. (E) R‐2HG decreased FTO and transcription factors expression in PLC^AMOE^ cells

Recently, m6A‐methylation has been proved as an important regulator in mRNA stability of pluripotency factors.^19^, ^23^ We next investigated whether AMD1 could affect m6A levels of mRNA in HCC cells. Compared with the corresponding control groups, overexpression of AMD1 in PLC significantly decreased the total m6A+ RNA levels, while knockdown of AMD1 in MHCC97H cells increased the total m6A+ RNA levels (Figure 5A). Then we conducted MeRIP‐seq of MHCC97H^AMKD^ cells with MHCC97H^AMNC^ cells. The results showed that there were 1877 hyper‐methylated peaks in MHCC97H^AMKD^ cells compared with control cells (Figure 5B). Furthermore, knockdown of AMD1 indeed increased the abundance of m6A in the 5′UTR and CDS regions of SOX2 and KLF4 transcripts (Figure 5C). By predicting m6A sites using SRAMP, a computational predictor of mammalianm6A site, we found that m6A sites were mainly distributed in the 5′UTR and CDS regions of NONOG, SOX2, and KLF4 transcripts. For OCT4, only one m6A site was predicted in 3′UTR regions (Figure 5D). Using gene‐specific m6A qPCR assays, we confirmed that high AMD1 expression in HCC cells decreased m6A level of NANOG CDs regions. However, there was no significant change in m6A modification in the 3′UTR regions of OCT4 transcripts (Figure 5E). We hypothesized AMD1 affected mRNA m6A levels through regulating the dynamic balances of methylation effects mediated by m6A methyltransferases and demethylases. The effects of AMD1 on METTL3, METTL14, ALKBH5, and FTO were analyzed. Results indicated that overexpression of AMD1 in PLC cells increased FTO protein levels, whereas AMD1 knockdown in MHCC97H and HCCLM3 cells decreased FTO protein levels. No significant roles of AMD1 on METTL3/METTL14/ALKBH5 were observed in protein levels (Figure 5F). The FTO mRNA level was not regulated by AMD1 expression (data not shown). Consistent with this finding, there existed a significant positive correlation between AMD1 and FTO protein levels in our HCC tissues (R = 0.7080, *p* <0.001, Figures 6A‐6D). Moreover, similar trend was observed in xenograft tumors (Figure 6E). IHC results also showed FTO expression was positively associated with rate of positive cells of NANOG, SOX2, and KLF4 in HCC samples in the TMA (Figures S1A and S1B). We also confirmed FTO as a cancerous gene with poor prognosis in our research (Figures 6F and 6G). Restoration of FTO expression resulted in the upregulation of tumor spheres formation rate and related transcriptional factors of MHCC97H^AMKD^ cells. Simultaneously, silencing FTO expression by siRNA resulted in a reverse effect in PLC^AMOE^ cells (Figures 7A‐7D). Moreover, R‐2‐hydroxyglutarate, a special FTO inhibitor, could abolished NANOG, SOX2, and KLF4 expression in PLC^AMOE^ cells (Figure 7E). Results above suggested that FTO was a downstream target of AMD1 in regulating HCC cells stemness.

### 3.6 SPD promoted FTO expression via polyamination of IQGAP1

**FIGURE 8:**
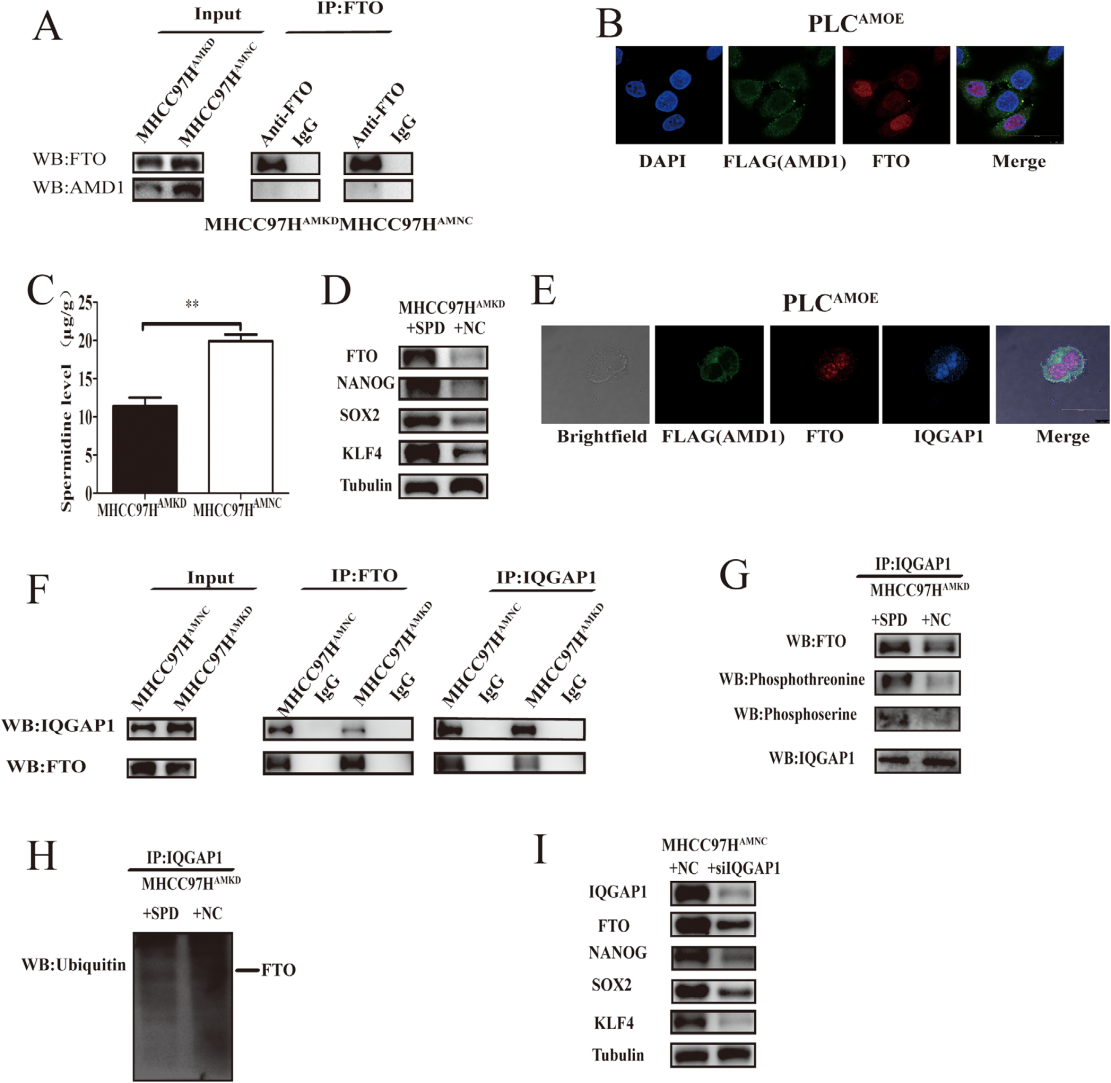
Spermidine promotes FTO expression via IQGAP1. (A and B) Co‐IP and immunofluorescence assays were used to detect the direct interaction of AMD1 and FTO. (C) Quantification of the spermidine level in MHCC97H cells transfected with AMD1 knockdown and the negative control vectors. **p < 0.005 (D) Western blot analysis was used to detect the expression change of FTO, NANOG, SOX2, and KLF4 on spermidine treatment. (E) Immunofluorescence assays were used to detect the direct interaction of FTO and IQGAP1. (F and G) Co‐IP and western blot analysis were used to evaluate the associativity of FTO and IQGAP1 and the phosphorylation of FTO on spermidine treatment. IQCAP1 was used as a loading control. (H) Ubiquitination assay for the effects of spermidine on FTO ubiquitination. (I) Western blot analysis was used to detect the expression change of FTO, NANOG, SOX2, and KLF4 on IQGAP1 deficient condition

The result of immunofluorescence and IP assay showed there was not an obvious interaction between AMD1 and FTO (Figures 8A and 8B), suggesting that AMD1 may regulate FTO expression through a roundabout way. AMD1 is a rate‐limiting enzyme in polyamines synthesis, which promotes the conversion of S‐adenosylmethionine to decarboxylated S‐adenosylmethionine. Putrescine is then converted to SPD and SPM sequentially with the donation of propylamine moiety from dcAdoMet.^33^ Indeed, high AMD1 protein levels could increase levels of the polyamine SPD in HCC cells (Figure 8C). To determine the role of SPD in regulating HCC cells FTO expression and stemness, MHCC97H^AMKD^ cells were treated with SPD. High level of SPD could upregulate FTO and pluripotency factors expression (Figure 8D). These results suggested that polyamine metabolism may affect HCC cells stemness through regulating FTO expression.

To elucidate the underlying mechanisms by which polyamines regulates FTO expression, IP and MS assays were performed to identify the post‐translational modification of FTO in MHCC97H^AMKD^ cells and control groups. Although FTO was not modified by polyamines directly, its phosphorylation decreased in MHCC97H^AMKD^ cells compared with MHCC97H^AMNC^ groups (Figures S2A‐S2C). Interestingly, we found that IQGAP1, a skeleton protein which overlapped both using the open search strategy and mascot search engine, interacted with FTO directly. This was also verified by immunofluorescence (Figure 8E). In addition, we found glutamine 372 (Q372) was the main polyamination site modified by SPD in IQGAP1 (Figure S3A). We verified this with western blot assay. The band of SPD could be detected in the position which was very close to IQGAP1 (Figure S3B). Further, high AMD1 expression enhanced the interaction between FTO and IQGAP1 (Figure 8F). Treatment for MHCC97H^AMKD^ cells with SPD indeed increased FTO phosphorylation and decreased FTO ubiquitylation (Figures 8G and 8H). Unsurprisingly, knockdown of IQGAP1 could efficiently decrease the expression of FTO, NANO, SOX2, and KLF4 (Figure 8I). These experiments indicated SPD as a putative upstream regulator stabilizing the interaction of IQGAP1 with FTO, then influencing FTO protein level.

## 4 DISCUSSION

AMD1 plays a key role in biosynthesis of SPD and SPM.^11^, ^12^ It is also found to be an oncogene in multiple cancers and a potential target for tumor therapy.^16^, ^17^, ^18^ However, up to now, there is no research about AMD1 effect on liver cancer, prompting us to investigate its oncological functions and clinical significances in HCC.

In this study, we first confirmed that the expression of AMD1 was significantly increased in HCC tumor tissues. Elevated AMD1 expression was significantly correlated with reduced OS and DFS of HCC patients. Moreover, intratumoral AMD1 level was an independent prognostic factor for both OS and DFS. In our samples, AMD1 expression of HCC tissues was positively correlated with the preoperative level of serum AFP. Serum AFP level is acknowledged as one of the most valuable serum tumor marker on diagnosis, evaluating prognosis and supervising recurrence of HCC.^34^ On the other hand, HCC cells with high AFP expression possessed stemness properties.^35^, ^36^ Recent studies showed that the number of circulating CSCs were significant positive correlated with serum AFP lever in HCC patients.^37^ Other common tumor markers such as ALDH and Glypican‐3 (GPC3) were also reported to express in HCC and correlated with HCC CSCs.^38^, ^39^, ^40^Although there are no clinical researches on AMD1 as a biomarker in HCC, some evidence still remains that the concentration of SPD in plasma differs significantly between HCC and lung cancer patients (at least 40 times).^41^ It means AMD1 and other enzymes participating in the polyamines metabolism may become potential candidate for differential diagnoses HCC with other cancers. Hence, all these results elucidated AMD1 as a potential biomarker for HCC diagnosis and prognosis, as well as, a novel potential target for HCC therapy.

Several reports have shown that AMD1 is essential for the maintenance of ESCs stemness. AMD1 could promote the expression of pluripotency factors such as OCT4, SOX2, and NANOG, then enhancing ESCs reprogramming.^14^, ^15^ In this study, overexpression of AMD1 in HCC cells could indeed result in NANOG, SOX2, and KLF4 upregulation. These stemness genes play central roles in HCC initiation, metastasis, and therapeutic resistance. Thus, the elimination of HCC cells stemness is an important therapeutic strategy to improve the prognosis of HCC patients.^42^ Our functional studies revealed that knockdown of AMD1 decreased HCC cells growth and metastasis in vivo. Sorafenib, an oral multikinase inhibitor, has become the first‐line treatment option for patients with advanced HCC. However, only a few patients can benefit from sorafenib.^43^ HCC CSCs are relatively resistant to sorafenib, and the high resistant rate has significantly limited the benefit of sorafenib therapy.^44^ In our study, knockdown of AMD1 could attenuate sorafenib resistance in HCC cells. SAM486, the specific inhibitor of AMD1, showed potent antitumor activity^17^, ^45^ and has been tested for safety and effectiveness in patients with a variety of tumors.^46^, ^47^, ^48^, ^49^ Furthermore, treatment with SAM486 could increase tumor differentiation.^17^ The AMD1 inhibitor may be an effective alternative agent in combination with sorafenib for the treatment of HCC. MINDY1, a deubiquitinase targeting Lysine 48 (K48) linked polyubiquitination specifically, was reported as a candidate target of AMD1 or polyamines in regulating ESCs stemness. But the exact mechanism is currently unclear. We also detected MINDY1 mRNA and protein levels in HCC cells and found its expression was not regulated by AMD1 observably (data not shown). This might stem from the difference of species and histologic type.^50^

As mentioned, demethylation of N6‐methyladenosine of mRNA of multiple pluripotency factors would increase the mRNA stability and then promote protein expression.^19^, ^20^ Therefore, we hypothesized that AMD1 also enhanced HCC cells stemness through regulating mRNA m6A levels. As shown in our research, AMD1 was found to increase FTO protein level in HCC cells, which has been proved to mediate m6A‐demethylation of mRNAs and play an oncogenic role in glioblastoma, acute myeloid leukemia, and HCC.^22^, ^51^, ^52^, ^53^, ^54^, ^55^ In our study, no significantly changes of OCT4 expression or m6A+ level was observed under regulation of AMD1. There was only one m6A site predicted in its 3′UTR regions. It was reported previously that FTO can increase the transcript levels of genes by reducing m6A abundance in the 5′UTR and CDs regions.^53^ Besides, overexpression of FTO in acute myeloid leukemia cells could decrease m6A modification of NANOG and SOX2 and increase their mRNA levels,^51^ which was consistent with our studies in HCC cells. In our study, restoration of FTO expression effectively antagonized the effects of AMD1 downregulation, whereas knockdown of FTO efficiently reversed the effects of AMD1 overexpression, suggesting that FTO may be the downstream target of AMD1 in these processes.

Prior studies have noted that polyamines are associated with colon carcinogenesis, and polyamine metabolism is a target for colon cancer chemoprevention.^56^, ^57^, ^58^ High polyamines level could promote ESCs and CSCs self‐renewal and maintain their stemness.^14^, ^15^, ^59^ Our study suggested that high AMD1 expression and intracellular SPD level could increase FTO levels mainly on post‐translational mechanisms indirectly. Polyamines were reported to be conjugated to proteins as a post‐translational modification covalently, which was defined as polyamination, leading to significant alters of conformations and functions of targets.^60^, ^61^, ^62^ Using IP and MS assays, we found IQGAP1, a scaffold protein, combined with FTO and was modified with SPD. Targeted MS analysis verified that the glutamine residue (Q) at position 372 was a primary modification site (Figure S3A). Two of the newly putative phosphorylation sites on FTO (T26 and S482) were identified by MS assay (Figures S2B and S2C). As consistent with our result, the phosphorylation of FTO could increase its overexpressing via suppressing the ubiquitination intracellularly.^63^ Polyamination was found to stabilize combinations of proteins,^61^, ^64^ and our research showed this modification indeed enhances the interaction between FTO and IQGAP1 (Figure 8G). IQGAP1 is a highly conserved scaffolding protein, which combines with multiple phosphokinases and modulates their signaling.^65^, ^66^, ^67^ It could promote tumor progression through increasing the phosphorylation of downstream proteins.^68^, ^69^ Our study showed that knockdown of IQGAP1 could efficiently decrease the expression of FTO, NANO, SOX2, and KLF4. Further research would be taken to elucidate the point of view.

## 5 CONCLUSIONS

**FIGURE 9:**
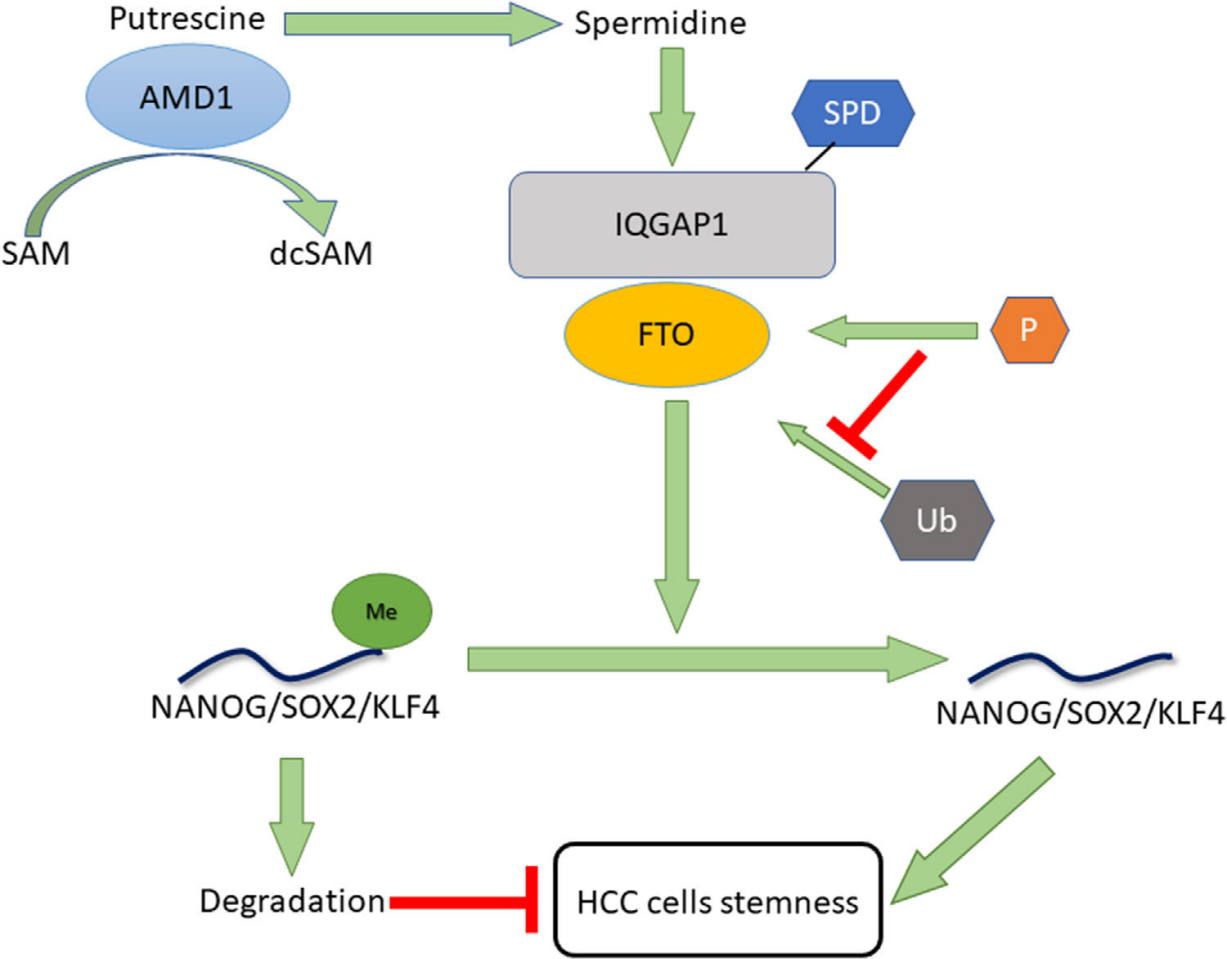
Mechanisms by which AMD1 promotes hepatocellular carcinoma cell stemness

In summary, our findings revealed that AMD1 was upregulated in HCC tissues and an adverse prognostic factor in HCC. AMD1 dramatically promoted HCC cells NANOG, SOX2, and KLF4 expression through FTO‐mediated mRNA demethylation. Mechanistically, high levels of AMD1 could increase the levels of SPD in HCC cells, which could modify the scaffold protein IQGAP1 and enhance the interaction between IQGAP1 and FTO. This interaction could enhance the phosphorylation and decrease the ubiquitination of FTO, thus increasing FTO protein expression (Figure 9). Our findings of AMD1 in regulating HCC cell stemness would not only broaden our knowledge but also provide a new strategy for HCC therapy in clinic.

## CONFLICT OF INTEREST

The authors declare that there is no conflict of interest that could be perceived as prejudicing the impartiality of the research reported.

## ETHICS APPROVAL AND CONSENT TO PARTICIPATE

All experiments were approved by the Ethics Committee of Zhongshan Hospital.

## AUTHOR CONTRIBUTIONS

Weizhong Wu, Xinyu Bian, Dongmin Shi, and Dahai Yu conceived and designed the study. Xinyu Bian, Dongmin Shi, and Kailin Xing performed the experiments. Xinyu Bian, Kailin Xing, Hongxin Zhou, and Lili Lu analyzed and interpreted data. Xinyu Bian, Dongmin Shi and Weizhong Wu wrote the manuscript. Weizhong Wu and Dahai Yu revised the paper and were the study supervisors. All authors read and approved the final manuscript.

## Supporting information

SUPPORTING INFORMATION

SUPPORTING INFORMATION

## Data Availability

The data used in the current study are available from the corresponding author on reasonable request.

## References

[1] Forner A , Reig M , Bruix J . Hepatocellular carcinoma. review. Lancet. 2018;391(10127):1301–1314. 29307467 10.1016/S0140-6736(18)30010-2

[2] Torre LA , Bray F , Siegel RL , Ferlay J , Lortet‐Tieulent J , Jemal A . Global cancer statistics, 2012. CA Cancer J Clin. 2015;65(2):87–108. 25651787 10.3322/caac.21262

[3] Boyer LA , Lee TI , Cole MF , et al. Core transcriptional regulatory circuitry in human embryonic stem cells. Cell. 2005;122(6):947–956. 16153702 10.1016/j.cell.2005.08.020 PMC3006442

[4] Valent P , Bonnet D , De Maria R , et al. Cancer stem cell definitions and terminology: the devil is in the details. Nat Rev Cancer. 2012;12(11):767–775. 23051844 10.1038/nrc3368

[5] Gupta PB , Chaffer CL , Weinberg RA . Cancer stem cells: mirage or reality?. Nat Med. 2009;15(9):1010–1012. 19734877 10.1038/nm0909-1010

[6] Visvader JE , Lindeman GJ . Cancer stem cells in solid tumours: accumulating evidence and unresolved questions. Nat Rev Cancer. 2008;8(10):755–768. 18784658 10.1038/nrc2499

[7] Liu Q , Chen K , Liu Z , et al. BORIS up‐regulates OCT4 via histone methylation to promote cancer stem cell‐like properties in human liver cancer cells. Cancer Lett. 2017;403:165–174. 28645561 10.1016/j.canlet.2017.06.017

[8] Chiba T , Kamiya A , Yokosuka O , Iwama A . Cancer stem cells in hepatocellular carcinoma: recent progress and perspective. Cancer Lett. 2009;286(2):145–153. 19464789 10.1016/j.canlet.2009.04.027

[9] Chiba T , Iwama A , Yokosuka O . Cancer stem cells in hepatocellular carcinoma: therapeutic implications based on stem cell biology. Hepatol Res. 2016;46(1):50–57. 26123821 10.1111/hepr.12548

[10] Kim HJ , Jeong J , Park S , et al. Establishment of hepatocellular cancer induced pluripotent stem cells using a reprogramming technique. Gut Liver. 2017;11(2):261–269. 27728962 10.5009/gnl15389 PMC5347651

[11] Pegg AE . S‐Adenosylmethionine decarboxylase. Essays Biochem. 2009;46:25–45. 20095968 10.1042/bse0460003

[12] Pegg AE . Mammalian polyamine metabolism and function. Iubmb Life. 2009;61(9):880–894. 19603518 10.1002/iub.230 PMC2753421

[13] Yu CH , Chou CC , Lee YJ , Khoo KH , Chang GD . Uncovering protein polyamination by the spermine‐specific antiserum and mass spectrometric analysis. Amino Acids. 2015;47(3):469–481. 25471600 10.1007/s00726-014-1879-8

[14] Zhang D , Zhao T , Ang HS , et al. AMD1 is essential for ESC self‐renewal and is translationally down‐regulated on differentiation to neural precursor cells. Genes Dev. 2012;26(5):461–473. 22391449 10.1101/gad.182998.111 PMC3305984

[15] Zhao T , Goh KJ , Ng HH , Vardy LA . A role for polyamine regulators in ESC self‐renewal. Cell Cycle. 2012;11(24):4517–4523. 23165208 10.4161/cc.22772 PMC3562295

[16] Zabala‐Letona A , Arruabarrena‐Aristorena A , Martin‐Martin N , et al. mTORC1‐dependent AMD1 regulation sustains polyamine metabolism in prostate cancer. Nature. 2017;547(7661):109–113. 28658205 10.1038/nature22964 PMC5505479

[17] Evageliou NF , Haber M , Vu A , et al. Polyamine antagonist therapies inhibit neuroblastoma initiation and progression. Clin Cancer Res. 2016;22(17):4391–4404. 27012811 10.1158/1078-0432.CCR-15-2539

[18] Ali HEA , Lung PY , Sholl AB , et al. Dysregulated gene expression predicts tumor aggressiveness in African‐American prostate cancer patients. Sci Rep. 2018;8(1):16335. 30397274 10.1038/s41598-018-34637-8 PMC6218553

[19] Batista PJ , Molinie B , Wang J , et al. m(6)A RNA modification controls cell fate transition in mammalian embryonic stem cells. Cell Stem Cell. 2014;15(6):707–719. 25456834 10.1016/j.stem.2014.09.019 PMC4278749

[20] Wang X , Lu Z , Gomez A , et al. N‐6‐methyladenosine‐dependent regulation of messenger RNA stability. Nature. 2014;505(7481):117–120. 24284625 10.1038/nature12730 PMC3877715

[21] Yue Y , Liu J , He C . RNA N6‐methyladenosine methylation in post‐transcriptional gene expression regulation. Genes Dev. 2015;29(13):1343–1355. 26159994 10.1101/gad.262766.115 PMC4511210

[22] Ma JZ , Yang F , Zhou CC , et al. METTL14 suppresses the metastatic potential of hepatocellular carcinoma by modulating N(6) ‐methyladenosine‐dependent primary MicroRNA processing. Hepatology. 2017;65(2):529–543. 27774652 10.1002/hep.28885

[23] Zhang C , Samanta D , Lu H , et al. Hypoxia induces the breast cancer stem cell phenotype by HIF‐dependent and ALKBH5‐mediated m(6)A‐demethylation of NANOG mRNA. Proc Natl Acad Sci U S A. 2016;113(14):E2047–E2056. 27001847 10.1073/pnas.1602883113 PMC4833258

[24] Li XH , Zhou XM , Li XJ , et al. Effects of xiaoyaosan on the hippocampal gene expression profile in rats subjected to chronic immobilization stress. Front Psychiatry. 2019;10:178. 31031647 10.3389/fpsyt.2019.00178 PMC6474260

[25] Bian X , Wu P , Sha H , et al. Anti‐EGFR‐iRGD recombinant protein conjugated silk fibroin nanoparticles for enhanced tumor targeting and antitumor efficiency. Onco Targets Ther. 2016;9:3153–3162. 27313461 10.2147/OTT.S100678 PMC4892850

[26] Zhang Y‐Y , Kong L‐Q , Zhu X‐D , et al. CD31 regulates metastasis by inducing epithelial–mesenchymal transition in hepatocellular carcinoma via the ITGB1‐FAK‐Akt signaling pathway. Cancer Lett. 2018;429:29–40. 29746931 10.1016/j.canlet.2018.05.004

[27] Liu Y , Liu DL , Dong LL , et al. miR‐612 suppresses stem cell‐like property of hepatocellular carcinoma cells by modulating Sp1/Nanog signaling. Cell Death Dis. 2016;7(9):e2377. 27685621 10.1038/cddis.2016.282 PMC5059880

[28] Yang ZF , Ho DW , Ng MN , et al. Significance of CD90+ cancer stem cells in human liver cancer. Cancer Cell. 2008;13(2):153–166. 18242515 10.1016/j.ccr.2008.01.013

[29] Fermin D , Basrur V , Yocum AK , Nesvizhskii AI . Abacus: a computational tool for extracting and pre‐processing spectral count data for label‐free quantitative proteomic analysis. Proteomics. 2011;11(7):1340–1345. 21360675 10.1002/pmic.201000650 PMC3113614

[30] Li D , Lu S , Liu W , Zhao X , Mai Z , Zhang G . Optimal settings of mass spectrometry open search strategy for higher confidence. J Proteome Res. 2018;17(11):3719–3729. 30265008 10.1021/acs.jproteome.8b00352

[31] Potter VR . Phenotypic diversity in experimental hepatomas: the concept of partially blocked ontogeny. The 10th Walter Hubert Lecture. Br J Cancer. 1978;38(1):1–23. 356869 10.1038/bjc.1978.159 PMC2009671

[32] Geula S , Moshitch‐Moshkovitz S , Dominissini D , et al. Stem cells. m6A mRNA methylation facilitates resolution of naive pluripotency toward differentiation. Science. 2015;347(6225):1002–1006. 25569111 10.1126/science.1261417

[33] Gerner EW , Meyskens FL . Polyamines and cancer: old molecules, new understanding. Nat Rev Cancer. 2004;4(10):781–792. 15510159 10.1038/nrc1454

[34] Ma WJ , Wang HY , Teng LS . Correlation analysis of preoperative serum alpha‐fetoprotein (AFP) level and prognosis of hepatocellular carcinoma (HCC) after hepatectomy. World J Surg Oncol. 2013;11:212. 23981851 10.1186/1477-7819-11-212 PMC3844510

[35] Yamashita T , Kitao A , Matsui O , et al. Gd‐EOB‐DTPA‐enhanced magnetic resonance imaging and alpha‐fetoprotein predict prognosis of early‐stage hepatocellular carcinoma. Hepatology. 2014;60(5):1674–1685. 24700365 10.1002/hep.27093 PMC4142120

[36] Yamashita T , Ji J , Budhu A , et al. EpCAM‐positive hepatocellular carcinoma cells are tumor‐initiating cells with stem/progenitor cell features. Gastroenterology. 2009;136(3):1012–1024. 19150350 10.1053/j.gastro.2008.12.004 PMC2828822

[37] Zahran AM , Abdel‐Rahim MH , Refaat A , et al. Circulating hematopoietic stem cells, endothelial progenitor cells and cancer stem cells in hepatocellular carcinoma patients: contribution to diagnosis and prognosis. Acta Oncol. 2020;59(1):33–39. 31478425 10.1080/0284186X.2019.1657940

[38] Chiba T , Suzuki E , Yuki K , et al. Disulfiram eradicates tumor‐initiating hepatocellular carcinoma cells in ROS‐p38 MAPK pathway‐dependent and ‐independent manners. PLoS One. 2014;9(1):e84807. 24454751 10.1371/journal.pone.0084807 PMC3890271

[39] Castelli G , Pelosi E , Testa U . Liver cancer: molecular characterization, clonal evolution and cancer stem cells. Cancers (Basel). 2017;9(9):127. 10.3390/cancers9090127 PMC5615342 28930164

[40] Chen X , Lingala S , Khoobyari S , Nolta J , Zern MA , Wu J . Epithelial mesenchymal transition and hedgehog signaling activation are associated with chemoresistance and invasion of hepatoma subpopulations. J Hepatol. 2011;55(4):838–845. 21334406 10.1016/j.jhep.2010.12.043 PMC3177032

[41] Xu H , Liu R , He B , Bi CW , Bi K , Li Q . Polyamine metabolites profiling for characterization of lung and liver cancer using an LC‐Tandem MS method with multiple statistical data mining strategies: discovering potential cancer biomarkers in human plasma and urine. Molecules. 2016;21(8):1040. 10.3390/molecules21081040 PMC6273014 27517900

[42] Wang N , Wang S , Li MY , et al. Cancer stem cells in hepatocellular carcinoma: an overview and promising therapeutic strategies. Ther Adv Med Oncol. 2018;10. 10.1177/1758835918816287. PMC6304707 30622654

[43] Nguyen K , Jack K , Sun W . Hepatocellular carcinoma: past and future of molecular target therapy. Diseases. 2015;4(1):1. 10.3390/diseases4010001 PMC5456309 28933381

[44] Xin HW , Ambe CM , Hari DM , et al. Label‐retaining liver cancer cells are relatively resistant to sorafenib. Gut. 2013;62(12):1777–1786. 23411027 10.1136/gutjnl-2012-303261 PMC6993136

[45] Koomoa DL , Borsics T , Feith DJ , et al. Inhibition of S‐adenosylmethionine decarboxylase by inhibitor SAM486A connects polyamine metabolism with p53‐Mdm2‐Akt/protein kinase B regulation and apoptosis in neuroblastoma. Mol Cancer Ther. 2009;8(7):2067–2075. 19584241 10.1158/1535-7163.MCT-08-1217 PMC2731875

[46] Paridaens R , Uges DR , Barbet N , et al. A phase I study of a new polyamine biosynthesis inhibitor, SAM486A, in cancer patients with solid tumours. Br J Cancer. 2000;83(5):594–601. 10944598 10.1054/bjoc.2000.1305 PMC2363502

[47] Pless M , Belhadj K , Menssen HD , et al. Clinical efficacy, tolerability, and safety of SAM486A, a novel polyamine biosynthesis inhibitor, in patients with relapsed or refractory non‐Hodgkin's lymphoma: results from a phase II multicenter study. Clin Cancer Res. 2004;10(4):1299–1305. 14977828 10.1158/1078-0432.ccr-0977-03

[48] van Zuylen L , Bridgewater J , Sparreboom A , et al. Phase I and pharmacokinetic study of the polyamine synthesis inhibitor SAM486A in combination with 5‐fluorouracil/leucovorin in metastatic colorectal cancer. Clin Cancer Res. 2004;10(6):1949–1955. 15041711 10.1158/1078-0432.ccr-02-0995

[49] Siu LL , Rowinsky EK , Hammond LA , et al. A phase I and pharmacokinetic study of SAM486A, a novel polyamine biosynthesis inhibitor, administered on a daily‐times‐five every‐three‐week schedule in patients with Advanced solid malignancies. Clin Cancer Res. 2002;8(7):2157–2166. 12114416

[50] James C , Zhao TY , Rahim A , et al. MINDY1 is a downstream target of the polyamines and promotes embryonic stem cell self‐renewal. Stem Cells. 2018;36(8):1170–1178. 29644784 10.1002/stem.2830

[51] Li Z , Weng H , Su R , et al. FTO plays an oncogenic role in acute myeloid leukemia as a N(6)‐methyladenosine RNA demethylase. Cancer Cell. 2017;31(1):127–141. 28017614 10.1016/j.ccell.2016.11.017 PMC5234852

[52] Zhang S , Zhao BS , Zhou A , et al. m(6)A demethylase ALKBH5 maintains tumorigenicity of glioblastoma stem‐like cells by sustaining FOXM1 expression and cell proliferation program. Cancer Cell. 2017;31(4):591–606. 28344040 10.1016/j.ccell.2017.02.013 PMC5427719

[53] Su R , Dong L , Li C , et al. R‐2HG exhibits anti‐tumor activity by targeting FTO/m(6)A/MYC/CEBPA signaling. Cell. 2018;172(1‐2):90–105 e23. 29249359 10.1016/j.cell.2017.11.031 PMC5766423

[54] Cui Q , Shi H , Ye P , et al. m(6)A RNA methylation regulates the self‐renewal and tumorigenesis of glioblastoma stem cells. Cell Rep. 2017;18(11):2622–2634. 28297667 10.1016/j.celrep.2017.02.059 PMC5479356

[55] Chen J , Du B . Novel positioning from obesity to cancer: FTO, an m(6)A RNA demethylase, regulates tumour progression. J Cancer Res Clin Oncol. 2019;145(1):19–29. 30465076 10.1007/s00432-018-2796-0 PMC11810187

[56] Gerner EW , Meyskens FL, Jr , Goldschmid S , Lance P , Pelot D . Rationale for, and design of, a clinical trial targeting polyamine metabolism for colon cancer chemoprevention. Amino Acids. 2007;33(2):189–195. 17396214 10.1007/s00726-007-0515-2

[57] Gerner EW . Combination chemoprevention for colon cancer targeting polyamine synthesis and inflammation. Clin Cancer Res. 2009;15(3):758–761. 19188144 10.1158/1078-0432.CCR-08-2235 PMC2666541

[58] Casero RA, Jr , Stewart TM , Pegg AE . Polyamine metabolism and cancer: treatments, challenges and opportunities. Nat Rev Cancer. 2018;18(11):681–695. 30181570 10.1038/s41568-018-0050-3 PMC6487480

[59] Tamari K , Konno M , Asai A , et al. Polyamine flux suppresses histone lysine demethylases and enhances ID1 expression in cancer stem cells. Cell Death Discov. 2018;4:104. 30455990 10.1038/s41420-018-0117-7 PMC6234213

[60] Song Y , Brady ST . Post‐translational modifications of tubulin: pathways to functional diversity of microtubules. Trends Cell Biol. 2015;25(3):125–136. 25468068 10.1016/j.tcb.2014.10.004 PMC4344850

[61] Song Y , Kirkpatrick LL , Schilling AB , et al. Transglutaminase and polyamination of tubulin: posttranslational modification for stabilizing axonal microtubules. Neuron. 2013;78(1):109–123. 23583110 10.1016/j.neuron.2013.01.036 PMC3627183

[62] Yu C‐H , Chou C‐C , Lee Y‐J , Khoo K‐H , Chang G‐D . Uncovering protein polyamination by the spermine‐specific antiserum and mass spectrometric analysis. Amino Acids. 2015;47(3):469–481. 25471600 10.1007/s00726-014-1879-8

[63] Tai H , Wang X , Zhou J , et al. Protein kinase Cbeta activates fat mass and obesity‐associated protein by influencing its ubiquitin/proteasome degradation. FASEB J. 2017;31(10):4396–4406. 28626026 10.1096/fj.201601159RR

[64] Baas PW . Microtubule stability in the axon: new answers to an old mystery. Neuron. 2013;78(1):3–5. 23583103 10.1016/j.neuron.2013.03.012

[65] White CD , Erdemir HH , Sacks DB . IQGAP1 and its binding proteins control diverse biological functions. Cell Signal. 2012;24(4):826–834. 22182509 10.1016/j.cellsig.2011.12.005 PMC3268868

[66] Goto T , Sato A , Adachi S , Iemura S , Natsume T , Shibuya H . IQGAP1 protein regulates nuclear localization of beta‐catenin via importin‐beta5 protein in Wnt signaling. J Biol Chem. 2013;288(51):36351–36360. 24196961 10.1074/jbc.M113.520528 PMC3868749

[67] Cheung KL , Lee JH , Shu L , Kim JH , Sacks DB , Kong AN . The Ras GTPase‐activating‐like protein IQGAP1 mediates Nrf2 protein activation via the mitogen‐activated protein kinase/extracellular signal‐regulated kinase (ERK) kinase (MEK)‐ERK pathway. J Biol Chem. 2013;288(31):22378–22386. 23788642 10.1074/jbc.M112.444182 PMC3829328

[68] Anakk S , Bhosale M , Schmidt VA , Johnson RL , Finegold MJ , Moore DD . Bile acids activate YAP to promote liver carcinogenesis. Cell Rep. 2013;5(4):1060–1069. 24268772 10.1016/j.celrep.2013.10.030 PMC3961013

[69] Hayashi H , Nabeshima K , Aoki M , et al. Overexpression of IQGAP1 in advanced colorectal cancer correlates with poor prognosis‐critical role in tumor invasion. Int J Cancer. 2010;126(11):2563–2574. 19856315 10.1002/ijc.24987

