# Supplementary material for: AMD1 upregulates hepatocellular carcinoma cells stemness by FTO mediated mRNA demethylation
Source: Clin Transl Med. 2021 Mar 24;11(3):e352. doi: 10.1002/ctm2.352 (PMC7989706; doi:10.1002/ctm2.352)
Supplement: Supplementary file 1 — SUPPORTING INFORMATION [file CTM2-11-e352-s001.pdf]

Figure S1

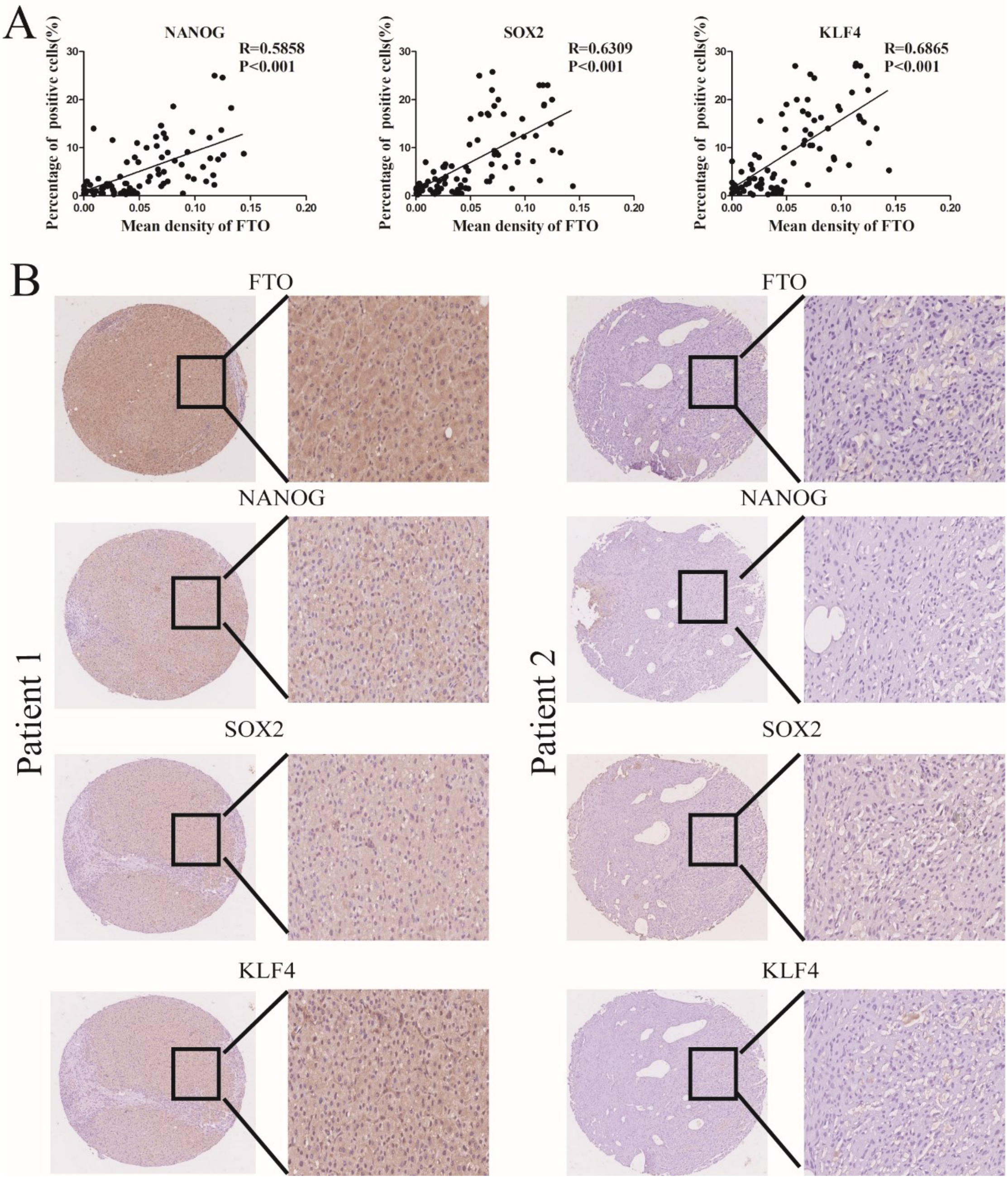

Figure S1. (A) Positive correlation between FTO and NANOG/SOX2/KLF4 in 85 HCC tissues. **(B-C)** Representative images of IHC staining of NANOG/SOX2/KLF4 in tumor tissues of HCC patients with high or low level of FTO expression.

Figure S2

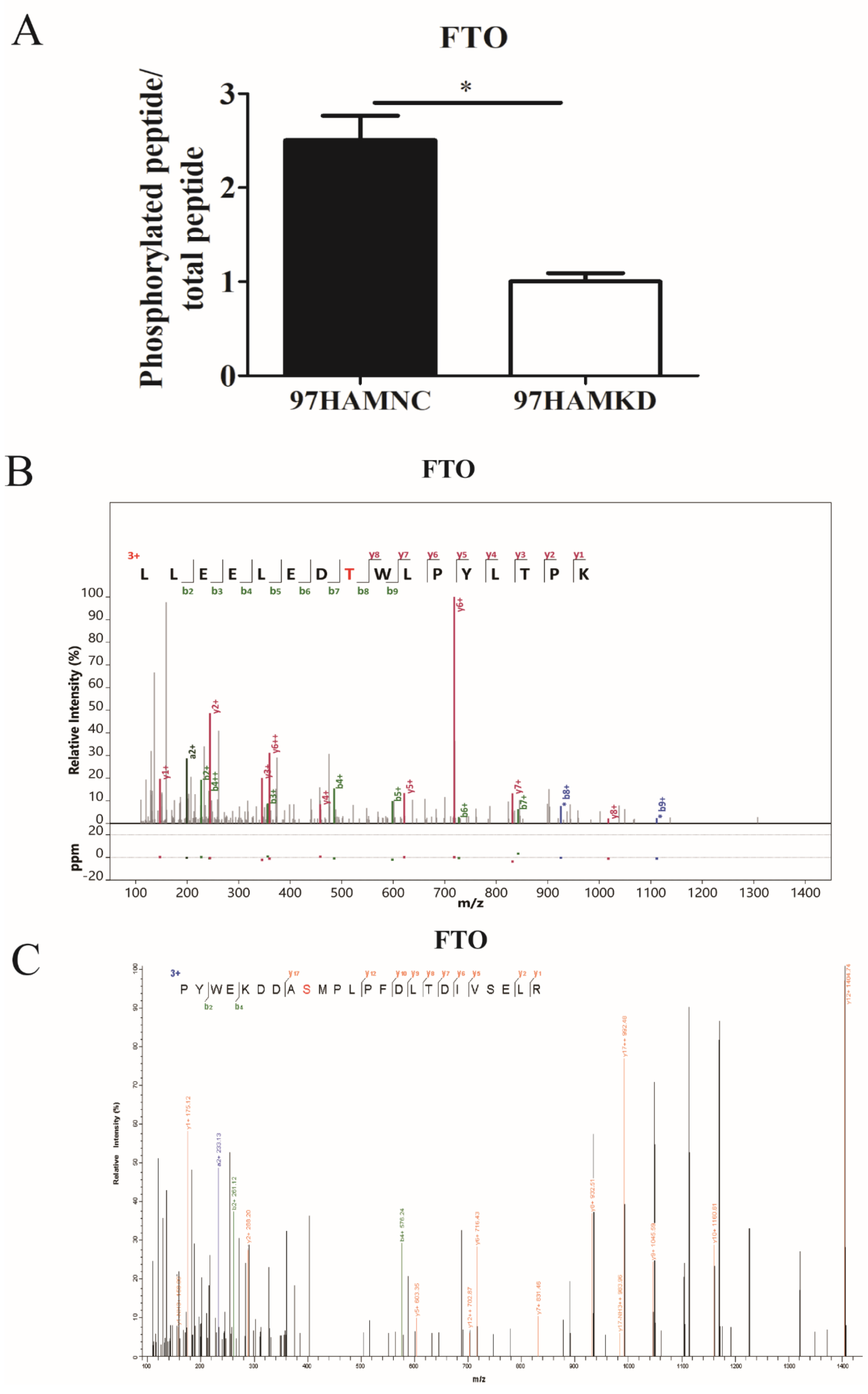
A
FTO
Phosphorylated peptide/ total peptide
*
97HAMNC
97HAMKD
B
FTO
3+
L L E E L E D T W L P Y L T P K
Relative Intensity (%)
ppm
m/z
C
FTO
3+
P Y W E K D D A S M P L P F D L T D I V S E L R
Relative Intensity (%)
m/z

Figure S2. (A) MS assay revealed FTO phosphorylation decreased in MHCC97H^{AMKD} cells compared with MHCC97H^{AMNC} groups. *P < 0.05. **(B-C)** Identification of the phosphorylation sites on FTO by a combination of Co-IP and MS.

Figure S3

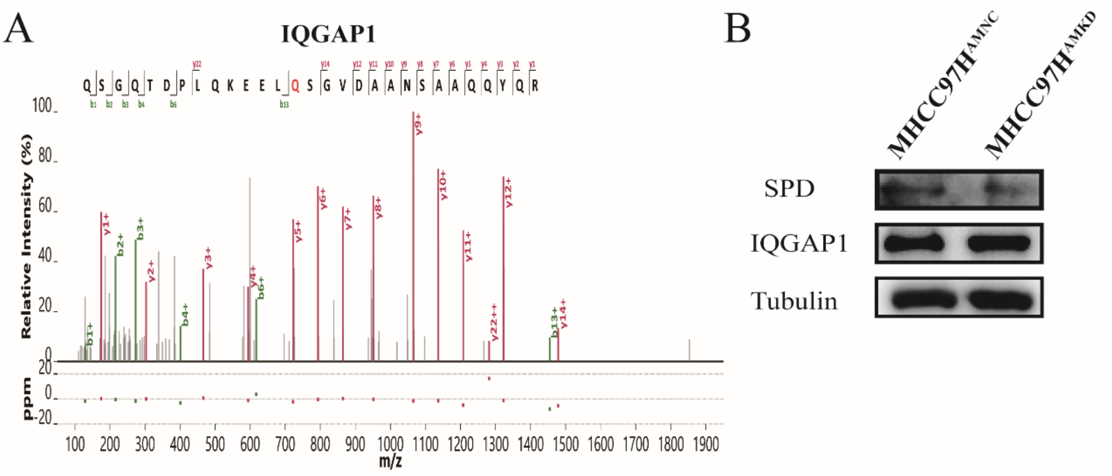

Figure S3. (A) Identification of the polyamination sites on IQGAP1 by a combination of Co-IP and MS. **(B)** Western blot assay was performed to detected bands of IQGAP1 and SPD in the same position.
