# Supplementary material for: AMD1 upregulates hepatocellular carcinoma cells stemness by FTO mediated mRNA demethylation
Source: Clin Transl Med. 2021 Mar 24;11(3):e352. doi: 10.1002/ctm2.352 (PMC7989706; doi:10.1002/ctm2.352)
Supplement: Supplementary file 2 — SUPPORTING INFORMATION [file CTM2-11-e352-s002.docx]

| Primers for qRT-PCR |  |  |
| --- | --- | --- |
| AMD1 | 5'-ACTCTCACGAGTGACATCCTTT-3' | 5'-AGTCGGGTAATCAGTCAGCCA-3' |
| NANOG | 5'-CCCCAGCCTTTACTCTTCCTA-3' | 5'-CCAGGTTGAATTGTTCCAGGTC-3' |
| SOX2 | 5'-ATCAGGAGTTGTCAAGGCAGAG-3' | 5'-AGAGGCAAACTGGAATCAGGA-3; |
| KLF4 | 5-'GATGATGCTCACCCCACCTT-3' | 5'-TGTGCCTTGAGATGGGAACTC-3' |
| FTO | 5'-TCCCAGTTACTCACCACACC3' | 5'-ACCGTCCAATACAGCAGTCA-3' |
| OCT4 | 5'-AAGCGATCAAGCAGCGAC-3' | 5'-GGAAAGGGACCGAGGAGTA-3' |
| GAPDH | 5'-ACAACTTTGGTATCGTGGAAGG-3' | 5'-GCCATCACGCCACAGTTTC-3' |
| 18S | 5'-CGGCGACGACCCATTCGAAC-3' | 5'-GAATCGAACCCTGATTCCCCGTC-3' |
|  |  |  |
|  |  |  |
| Primers for qRT-PCR after Merip | |  |
| NANOG | 5'-ACAAGGTCCCGGTCAAGAAA-3' | 5'-TGGAGGCTGAGGTATTTCTGT-3' |
| OCT4 | 5'-ATTCAAACTGAGGTGCCTGC-3' | 5'-TTGTGTTCCCAATTCCTTCCT-3' |
|  |  |  |
|  |  |  |
| Sequence of siRNA |  |  |
| si-FTO | 5'-AAAUAGCCGCUGCUUGUGAGA-3' |  |
| si-IQGAP1 | 5′-UUAUCGCCCAGA AACAUCUUGUUGG-3′ |  |
| si-NC | 5 -CAGGGTATCGACGATTACAAA-3 |  |

**Table S1. Primer and siRNA sequences**
